# Modular Chemical Descriptor Language (MCDL): Stereochemical modules

**DOI:** 10.1186/1758-2946-3-5

**Published:** 2011-01-31

**Authors:** Andrei A Gakh, Michael N Burnett, Sergei V Trepalin, Alexander V Yarkov

**Affiliations:** 1 Oak Ridge National Laboratory, Oak Ridge, Tennessee 37831, USA; 2 Institute Physiologically Active Compounds, Russian Academy of Sciences, 142432, Chernogolovka, Moscow region, Russia

## Abstract

**Background:**

In our previous papers we introduced the Modular Chemical Descriptor Language (MCDL) for providing a linear representation of chemical information. A subsequent development was the MCDL Java Chemical Structure Editor which is capable of drawing chemical structures from linear representations and generating MCDL descriptors from structures.

**Results:**

In this paper we present MCDL modules and accompanying software that incorporate unique representation of molecular stereochemistry based on Cahn-Ingold-Prelog and Fischer ideas in constructing stereoisomer descriptors. The paper also contains additional discussions regarding canonical representation of stereochemical isomers, and brief algorithm descriptions of the open source LINDES, Java applet, and Open Babel MCDL processing module software packages.

**Conclusions:**

Testing of the upgraded MCDL Java Chemical Structure Editor on compounds taken from several large and diverse chemical databases demonstrated satisfactory performance for storage and processing of stereochemical information in MCDL format.

## Background

In our previous paper we introduced the Modular Chemical Descriptor Language (MCDL) for providing a linear representation of structural and other chemical information [1]. All MCDL descriptors have two unique modules that describe the composition and the connectivity of a molecule. Optional supplementary modules, which may or may not be unique, contain additional information about a compound (such as spectra, physical-chemical data, atomic coordinates, references, *etc.*). The MCDL rules were implemented in the LINDES computer program, which was designed to generate MCDL linear descriptors from files containing molecular structural information in the form of a connectivity matrix.

A subsequent development was the MCDL Java Chemical Structure Editor which is capable of drawing chemical structures from linear representations and generating MCDL descriptors from structures [2]. Since the module containing atomic coordinates is an optional feature of MCDL descriptors, it was necessary for the Java applet to be capable of restoring these coordinates to draw a structure. As a result, the current applet algorithm that was developed to process MCDL descriptors can also be used for processing of other coordinate-less structure representations, such as SMILES [3,4] and InChI [5].

The initial MCDL concept [1] had one serious drawback - it did not support stereochemistry. In this paper we present optional MCDL modules and accompanying software that incorporate unique representation of molecular stereochemistry. The paper also contains some additional discussions regarding canonical representation of stereochemical isomers in MCDL format, and brief algorithm descriptions of the open source LINDES, Java applet, and Open Babel MCDL processing module software packages. The results of software testing are presented in the last section of the paper.

## Results and Discussion

### MCDL stereochemistry descriptors - theory

As noted previously [1], all MCDL linear descriptors include two primary modules that uniquely describe the basic molecular structure: the composition and the connectivity modules. The connectivity module is based on molecular topology, which adequately describes the sequence of bonds that connect atoms in the molecule. However, the topology-based connectivity module is inadequate for describing the three-dimensional arrangement of those atoms, which is the distinguishing characteristic in the structures of stereoisomers [see Appendix 1]. Refinement in the molecular structure representation in the MCDL can be achieved by employing a set of supplemental stereochemistry descriptors. The task is complicated by the existence of many types of stereoisomers [6,7]. The simplest are the common "optical" isomers of compounds with asymmetric atoms and the *cis*-*trans *isomers of compounds with double bonds. Less-common types of stereoisomers with more complex stereogenic units include "phase" isomers found in gear-like molecules [8,9] and chiral molecular knots [10,11]. In addition, a combination of different stereochemical types within a molecule makes comprehensive stereochemical analysis convoluted and often ambiguous. As a result, complete and unique representation of molecular stereochemistry is a compelling challenge.

Due to the complexity of underlying phenomena, specification of stereochemical information in the MCDL is currently limited to the two most common types - stereochemistry of a chiral atom in the {SA:} module and stereochemistry of a double bond in the {SB:} module. Within each type, a canonicalization procedure (described below) has been developed to generate unique stereodescriptors.

Systematic nomenclature of stereoisomers dates back to the pioneering works of Fischer [12,13] and Cahn-Ingold-Prelog (CIP) [14,15], all utilizing various schemes for a unique (canonical) prioritization of substituents attached to either an atomic center or to the two atoms connected by a double bond. The latter, and the most developed, CIP scheme uses atomic numbers as the basis for substituent priority ranking and requires sophisticated multi-level priority algorithms in the case of substituents with identical atomic numbers. CIP rules are known to produce ambiguous results due to the non-unique ranking of substituents in some complicated cases and have been under development during the last decades [16,17]. Nevertheless, the rules work relatively well for the majority of simple organic molecules.

The MCDL employs both CIP and Fischer ideas in constructing stereoisomer descriptors. Similar to CIP, the MCDL stereochemistry descriptors are based on prioritization of substituents, but unlike CIP, the MCDL algorithm uses planes, not axes, to specify the configuration of an atomic center (Fischer's approach). Although the algorithm rules are close to the CIP rules (priority ranking) [14,15], the resulting MCDL descriptors are not identical to the *R*-*S *and *E*-*Z *naming conventions due in large part to the differences in the underlying prioritization approaches.

Stereoisomer descriptors are expected to be unique in all cases where canonical numbering can be implemented. It is important to note that in cases where two or more constitutionally equivalent numbering schemes can be derived, all must be taken into consideration for the selection of the unique stereochemistry descriptor. This approach is currently the only reliable method for establishing the unique (canonical) descriptors and is very similar to one that has been developed previously for the unique MCDL connectivity modules [1].

### Atom stereochemistry (chiral centers)

Atom stereochemistry takes into consideration the three-dimensional arrangement of substituents around an atomic chiral center. In the majority of cases it is a four-coordinated atom (such as a carbon atom), but there is a substantial number of stereoisomers having chiral centers with three substituents. Notable examples include chiral sulfoxides.

The priorities of the substituents attached to a chiral center are the MCDL priorities (based on ASCII codes) of the attached fragments and terminal atoms, if any (see below). Once these are known, a Fischer projection of the configuration at the chiral atom is drawn. A Fischer projection is a planar representation of a molecule that preserves information about chirality. With the chiral atom at its center, a horizontal line represents two bonds bending forward toward the viewer, and a vertical line represents two bonds bending back away from the viewer. In the projection, the highest MCDL priority substituent on the chiral atom is placed at the top and the second highest at the bottom. The other two substituents appear at the left and right and are positioned to preserve the configuration of the chiral atom. Once oriented in this way, the atom stereochemistry is specified in the MCDL linear descriptor as {SA:chiral fragment,top,bottom,left,right} where the four positions refer to the positions in the Fischer projection.

#### One chiral center

**Figure 1:**
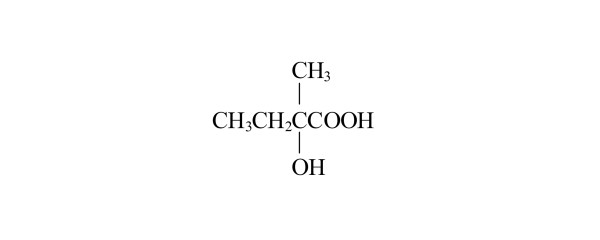
**Structural formula of 2-hydroxy-2-methylbutanoic acid**.

**Figure 2:**
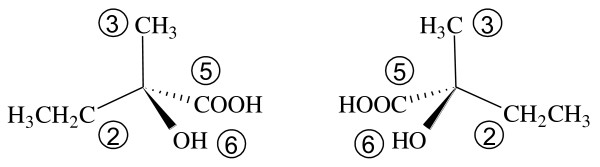
**Three-dimensional representations of 2-hydroxy-2-methylbutanoic acid's enantiomers with the MCDL priorities of the substituents on the chiral atom indicated**.

As an example, consider 2-hydroxy-2-methylbutanoic acid shown in Figure 1. The α-carbon is chiral, and thus this molecule has two mirror-image structures. The MCDL composition and connectivity modules for both of these are C;CHH;2CHHH;CO;2OH[2,3,5,6;4;;;7]. The chiral atom is fragment 1 in the descriptor. Its substituents and their priorities are CHH (2), CHHH (3), CO (5), and OH (6). Three-dimensional representations of this compound's enantiomers are shown in Figure 2 with the priorities of the substituents indicated.

**Figure 3:**
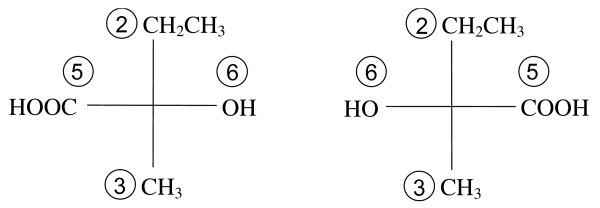
**Fischer projections of 2-hydroxy-2-methylbutanoic acid's enantiomers**.

The two structures are redrawn as Fischer projection formulas in Figure 3. The four substituents have been placed on the projection so the fragment having the highest priority is at the top and the next highest at the bottom. Thus, the stereochemistry for the left structure is specified in the MCDL as {SA:1,2,3,5,6} and as {SA:1,2,3,6,5} for the right structure.

**Figure 4:**
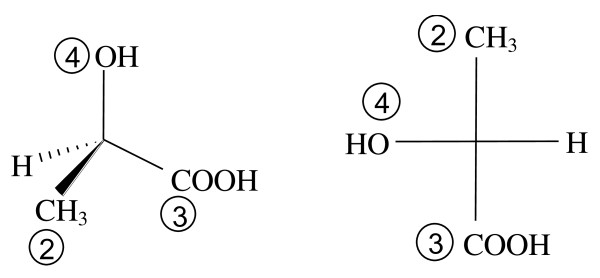
**Three-dimensional representation and Fischer projection of D-(*R*-)lactic acid**.

The example of D-lactic acid (*R*-lactic acid), shown in Figure 4, brings up a new issue to consider. The MCDL composition module of lactic acid is CH;CHHH;CO;2OH, and the chiral carbon is part of the first fragment. The attached fragments and their MCDL priorities are CHHH (2), CO (3), and OH (4). The fourth substituent on the chiral carbon, H, is actually part of fragment 1, but it must be considered on its own in order to specify the stereochemistry in the linear descriptor. To handle this situation, a new rule is added to the MCDL: Structural fragments have higher priorities than terminal atoms (numbers have lower ASCII codes than letters). Thus, of the four substituents attached to the chiral atom in lactic acid, the terminal atom H has the lowest priority. Orienting the *R *configuration as the Fischer projection in Figure 4 gives the MCDL descriptor {SA:1,2,3,4,H}.

#### Multiple chiral centers

The MCDL representation of multiple chiral centers in a molecule consists of a sequence of atomic configuration descriptors (one for each of the chiral centers), listed in descending priority order of the chiral fragments (smallest ASCII value first) and separated by semicolons. While treatment of structures with multiple chiral centers in the MCDL is straightforward in many cases, the presence of multiple chiral centers in certain quasi-symmetrical structures may lead to complications due to the fact that the unique part of the MCDL linear descriptor is generated without consideration of stereochemistry. As a result, constitutionally, but not stereochemically, equivalent chiral centers may receive arbitrarily selected fragment numbers.

**Figure 5:**
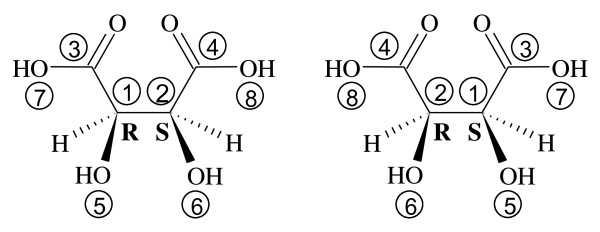
**Two possible MCDL numbering schemes for *meso*-tartaric acid**.

Figure 5 shows the 3-dimensional structure of *meso*-tartaric acid having two constitutionally identical, but stereochemically different, chiral centers (opposite configurations). Because of the topological symmetry of the molecule, two MCDL numbering schemes are possible. (If the chiral centers had the same configuration, the two numbering schemes would be identical.) The difference can be seen in Figure 5 in which either structural fragment 1 has the *R *configuration (left structure) or fragment 2 is *R *(right structure).

**Figure 6:**
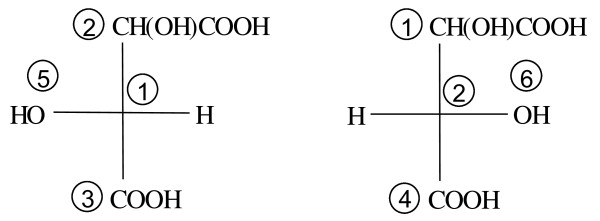
**The Fischer projections centered at fragments 1 and 2, respectively, of the left structure in Figure 5**.

Figure 6 shows the Fischer projections centered at fragments 1 and 2, respectively, of the left structure in Figure 5. The MCDL stereochemistry descriptor for this numbering scheme is {SA:1,2,3,5,H;2,1,4,H,6}.

**Figure 7:**
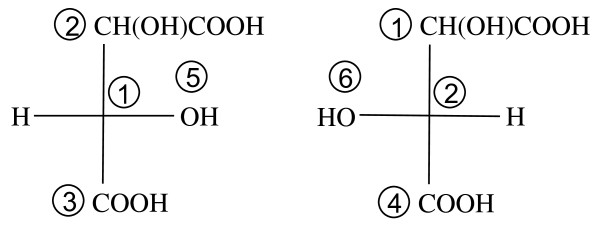
**The Fischer projections centered at fragments 1 and 2, respectively, of the right structure in Figure 5**.

Figure 7 corresponds to the right structure in Figure 5, giving the MCDL descriptor {SA:1,2,3,H,5;2,1,4,6,H}.

To choose the correct and unique stereodescriptor in cases like this, the two possible descriptors are compared position-by-position starting at the left. In this instance ({SA:1,2,3,5,H;2,1,4,H,6} vs. {SA:1,2,3,H,5;2,1,4,6,H}), the first difference occurs in the fourth position where one has a 5 and the other has an H. Since 5 is a higher priority than H, this difference allows us to choose {SA:1,2,3,5,H;2,1,4,H,6} as the MCDL stereochemistry descriptor for *meso*-tartaric acid. As a general rule, for quasi-symmetrical structures where multiple equivalent numbering schemes are possible, all must be explored for selection of the canonical (the lowest ASCII code sequence) stereochemistry descriptor. An alternative approach entails the use of hash variables for constitutionally equivalent stereogenic fragments (see *Generation of atomic MCDL stereodescriptors from structure diagrams *section below).

#### Three-substituent chiral centers

**Figure 8:**
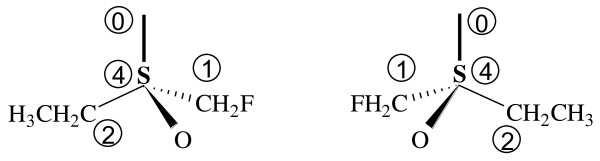
**Three-dimensional representations of ethyl(fluoromethyl)sulfoxide's enantiomers**. The "dummy" substituent is numbered as 0.

The MCDL can also specify chirality of atomic centers with only three substituents in those cases where the fourth substituent position is occupied by an electron pair. In this case, the electron pair is treated as a "dummy" substituent positioned at the point of maximum distance from the three "real" substituents on a sphere of unit radius centered at the chiral atom. The priority of the electron pair is defined in the MCDL as 0 (zero), giving it, maybe surprisingly, higher priority than structural fragments and terminal atoms. For example, the chirality descriptors of ethyl(fluoromethyl)sulfoxide (CFHH;CHH;CHHH;SO[4;3,4]), shown in Figure 8, are {SA:4,,1,2,O} for the left structure and {SA:4,,1,O,2} for the right structure. Note that the "dummy" 0-numbered substituent is not included in the descriptor.

### Double bond stereochemistry

The configuration of a double bond can be specified after the priorities of the structural fragments making up the molecule are known. Four items of information are needed:

• the priority numbers of the two fragments containing the double-bonded atoms - (***x***_***1***_, ***x***_***2***_, *x*_*1 *_<*x*_*2*_)

• the higher priority connection to the higher priority fragment of the double bond, *x*_*1 *_- (***n***_***1***_)

• the connection to the lower priority fragment of the double bond, *x*_*2*_, that lies on the *same side *of the double bond as *n*_*1 *_- (***n***_***2***_)

The stereochemistry is specified as {SB:*x*_*1*_d*x*_*2*_,*n*_*1*_,*n*_*2*_,*n*_*3*_,*n*_*4*_}, where *n*_*3 *_and *n*_*4 *_are the remaining two fragments attached to *x*_*2 *_and *x*_*1*_, respectively. Multiple double bond configurations are separated by semicolons and are listed with increasing values of *x*_*1*_.

#### One double bond

**Figure 9:**
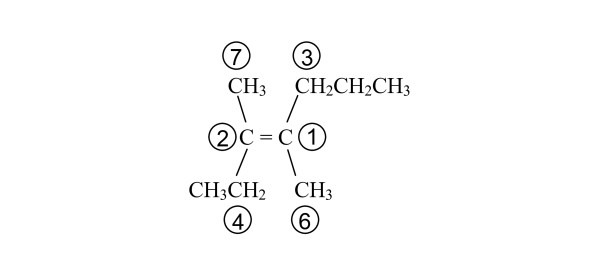
**MCDL numbering of 3,4-dimethyl-3-heptene for stereochemistry descriptor generation**.

As an example, consider 3,4-dimethyl-3-heptene with the configuration shown in Figure 9. The priorities of the fragments containing the double-bonded atoms and their immediate connections are shown. In this figure the priority numbers of the two fragments containing the double-bonded atoms are 1 and 2. Of the two connections (3 and 6) to the higher priority fragment of the double bond (1), fragment 3 has the higher priority. The connection to fragment 2 that lies on the same side of the double bond as fragment 3 is fragment 7. Thus, the stereochemistry of the compound in Figure 9 is specified as {SB:1d2,3,7,4,6}. Including the composition and connectivity modules, the MCDL linear descriptor is 2C;3CHH;4CHHH[2,3,6;4,7;5;8;9]{SB:1d2,3,7,4,6}.

**Figure 10:**
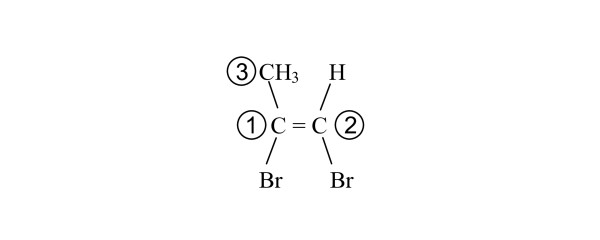
**MCDL numbering of 1,2-dibromopropene for stereochemistry descriptor generation**.

In the specification, the values of *n*_*1*_, *n*_*2*_, *n*_*3 *_and *n*_*4 *_are not necessarily structural fragment numbers. This can occur when connections to the double-bonded atoms are terminal atoms rather than structural fragments. Consider 1,2-dibromopropene in the configuration shown in Figure 10.

This compound consists of three structural fragments: CBr, CBrH, and CHHH. These have the MCDL priorities shown in the Figure 10. The priority numbers of the two fragments containing the double-bonded atoms are 1 and 2. Of the two connections (3 and Br) to the higher priority fragment of the double bond (1), fragment 3 has the higher priority. The connection to fragment 2 that lies on the same side of the double bond as fragment 3 is terminal atom H. The stereochemistry is thus specified {SB:1d2,3,H,Br,Br}. The complete MCLD descriptor is CBr;CBrH;CHHH[2,3]{SB:1d2,3,H,Br,Br}.

#### Multiple double bonds

**Figure 11:**
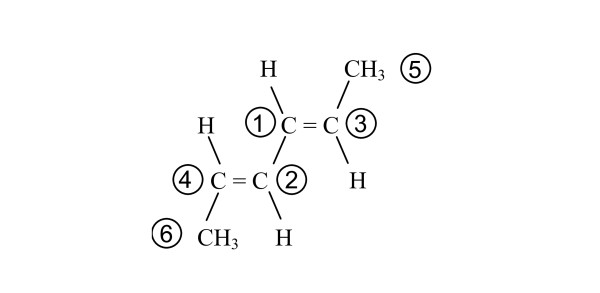
***Trans*,*trans*-hexa-2,4-diene**.

The MCDL representation of multiple double bonds in a molecule consists of a sequence of double bond configuration descriptors (one for each of the double bonds), listed in descending priority order of the higher priority member of the double bond (smallest ASCII value first) and separated by semicolons. As in the case of compounds with multiple chiral centers, the generation of a unique double bond descriptor for compounds with multiple double bonds having certain symmetry elements can be complicated. For example, consider hexa-2,4-diene. The unique portion of this diene's linear descriptor is 4CH;2CHHH[2,3;4;5;6] regardless of the configurations of the double bonds. The *trans*,*trans *diene (Figure 11) has only one possible numbering scheme, and its stereochemistry module is {SB:1d3,2,H,5,H;2d4,1,H,6,H}.

**Figure 12:**
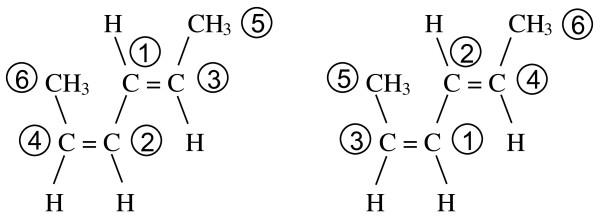
**Two alternative MCDL numbering schemes for *cis*,*trans*-hexa-2,4-diene**.

In contrast, the fragment priorities of *cis*,*trans*-hexa-2,4-diene can be assigned in two ways as shown in Figure 12. In the left drawing, the *trans *configuration occurs on the double bond between fragments 1 and 3 while in the right drawing the *trans *double bond is between fragments 2 and 4.

The two different numbering schemes give rise to stereochemistry specifications of {SB:1d3,2,H,5,H;2d4,1,6,H,H} and {SB:1d3,2,5,H,H;2d4,1,H,6,H}, respectively. To determine the correct descriptor, the *x *and *n *values of the two candidates are compared to each other beginning at the left. At the first point of difference, the descriptor having the higher priority *x *or *n *value is the correct one. The first difference in the two descriptors occurs at the third position where one has a structural fragment with priority value 5 and the other has the terminal atom H. As stated earlier, the structural fragment has a higher priority than the terminal atom. Thus, the correct stereochemistry descriptor is {SB:1d3,2,5,H,H;2d4,1,H,6,H}.

#### Two- or three-substituent double bonds

**Figure 13:**
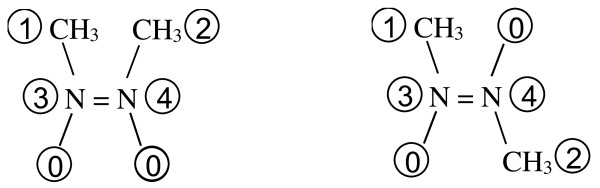
**MCDL numbering of *cis*- and *trans*-diaza-2-butene for stereochemistry descriptor generation. "Dummy" substituents have the highest priority (0)**.

Similar to atomic stereochemistry, MCDL double bond stereochemistry descriptors can be used for compounds containing three or two substituents attached to a double bond. In this case, absent substituents are replaced by "dummy" substituents having highest priority (0). Figure 13 provides the examples of *cis*- and *trans*-diaza-2-butene. The stereochemistry descriptor for *cis*-diaza-2-butene is {SB:3d4,,,2,1}, and for *trans*-diaza-2-butene it is {SB:3d4,,2,,1}. Again note the absence of the digits for "dummy" substituents.

### Mixed stereogenic units (atoms and bonds)

Molecules containing both stereogenic atoms and stereogenic bonds represent a special case for the MCDL. To ensure the uniqueness of the stereochemical descriptor in this case, the following rule has been added: atoms have higher priority than bonds. A similar approach has been implemented in the recently introduced InChI chemical descriptors [18]. Therefore, when multiple MCDL numbering schemes are possible for these compounds, first consideration should be given to schemes that provide the highest priorities to atomic chiral centers after which the priorities of atoms in double bonds are considered. All possible numbering schemes should be considered during this iterative process (see above).

In general, the same principle applies for molecules containing more complex stereogenic units (e.g., a double bond stereogenic unit with two atoms is higher priority than an allene-type stereogenic unit with three atoms - see additional file 1). Unfortunately, the complete classification of higher order stereogenic units is not possible in the framework of the current effort, so unique assignment of stereoisomers containing multiple instances of these units cannot be completed at the present time.

### Software implementation

#### LINDES 2.8

##### Stereochemistry algorithm

The major addition in the new release of the MCDL accompanying software, LINDES (version 2.8, see additional file 2) is the algorithm for recognition and generation of stereochemistry descriptors.

**Figure 14:**
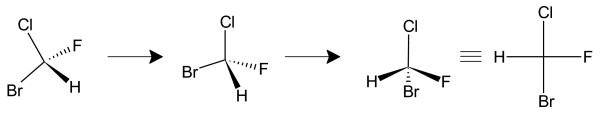
**Rotation of CHBrClF stereoisomer to give Fischer projection orientation**.

The software can determine the stereochemistry of a chiral atom in a molecule if the input consists of a MOLFILE having 3D coordinates or else 2D coordinates with *up *and *down *bond designations for the chiral atom(s) [19]. The algorithm first orients the molecule so the substituents on the stereogenic atom are positioned as they would appear in a Fischer projection. This is accomplished by rotating the molecule (fragment) until one substituent on the chiral atom is aligned with the +*y *axis and a second lies in the *yz *plane and has a negative *z *coordinate. The positions of the remaining two substituents are then identified by the signs of their *x *coordinates. This process is demonstrated in Figure 14, where the stereoisomer of CHBrClF shown at the left is rotated twice to give the orientation from which a Fischer projection can be drawn.

**Figure 15:**
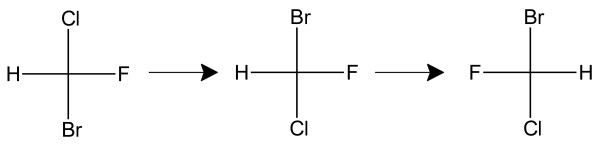
**Atom switches in Fischer projection of CHBrClF stereoisomer to maintain configuration**.

To determine the MCDL stereochemistry, the highest priority atom or fragment must be up, and the second highest must be down. In the present case, this can be accomplished by switching the positions of the Cl and Br. However, to maintain the same configuration as in the original Fischer projection, an EVEN number of switches is required. Thus, the H and F must also be switched (Figure 15). The final descriptor for this stereoisomer is {SA:1,Br,Cl,F,H}.

The software algorithm for determination of double bond stereochemistry works similarly to that described for chiral centers. The coordinates of the two double bonded atoms are needed along with the coordinates of one substituent on each of these. The double bond is oriented along the *y *axis and rotated to bring one of the substituents into the *xy *plane. The relative positions of the two substituents are identified by the signs of their *x *coordinates.

##### Software limitations

Version 2.8 of LINDES is designed to handle molecules with "simple" stereochemistry: one or more stereocenters, one or more double bonds, or a combination of these. To avoid problems associated with molecular symmetry, the current implementation requires the four atoms and/or MCDL fragments **directly **attached to the chiral atom or double bond to differ (i.e., the difference can not occur in a substituent at a point removed from the stereogenic center). For example, the substituents CH_2_Br (MCDL fragment CBrHH) and CH_2_Cl (MCDL fragment CClHH) are recognized as different while CH_2_CH_3 _and CH_2_CH_2_CH_3 _are not since the MCDL fragment at the point of attachment is CHH in both cases. An exception is made for molecules containing chiral CH fragments (such as sugars). The stereochemistry of double bonds within rings is ignored. LINDES 2.8 does not determine the stereochemistry of chiral centers or double bonds with fewer than four substituents. In specific cases, stereochemistry descriptors can be generated with LINDES by adding "dummy" substituents (see discussion above).

As stated earlier, for symmetrical and quasi-symmetrical structures where multiple constitutionally equivalent MCDL numbering schemes are possible, all must be explored for selection of the unique (the lowest ASCII code sequence) stereochemistry descriptor. LINDES 2.8 does not perform this exhaustive search.

LINDES 2.8 is not designed to handle the disjointed structures of mixtures (e.g., salts). To create the MCDL descriptors of salts and other mixtures, each component must be drawn and processed independently [see Appendix 2], and the resulting component descriptors have to be combined according to MCDL rules. Also, LINDES software does not process MOLFILEs that have no bond block.

### MCDL Java Chemical Structure Editor

#### Generation of structure diagrams from atomic MCDL stereodescriptors

**Figure 16:**
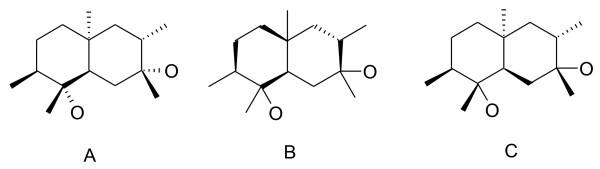
**Different depictions of the stereochemistry in an alicyclic molecule with chiral centers**.

If the stereoconfiguration of an atom is present in an MCDL descriptor, then it is reflected in the structure diagram using *up *and *down *bonds. The placement of these bonds is based on the criteria of simplicity, ease of understanding, and neat appearance of the resulting structure diagrams. The following rules have been developed to facilitate this task:

1. The maximum number of stereobonds attached to any particular atom should not exceed two. Stereobonds take a lot of space on the structure diagram, and it can become "overloaded" if more than two bonds are used to describe a chiral center (Figure 16, A).

2. If the chiral atom is a part of a ring, a bond outside the ring is chosen to receive the *up *or *down *attribute, if possible (Figure 16, B and 16C). If there are no exocyclic bonds, then a bond within the ring is chosen. It is also desirable to choose a cyclic bond that has the minimal number of substituents (Figure 16, C).

Once a bond is selected for stereo representation, it is then necessary to determine whether the *up *or *down *attribute reflects the proper stereoconfiguration provided in the MCDL stereodescriptor. To solve this problem, the algorithm initially selects *up *for the attribute and then re-creates the MCDL stereodescriptor of the particular chiral center. If the re-created descriptor is different from the original, the attribute is changed to *down*.

The MCDL Java applet does not explicitly draw the positions of hydrogen atoms in its structure diagrams. However, in many cases *up *and *down *stereobonds terminated by hydrogen atoms are used to display the stereoconfiguration of a chiral atom. These situations require transfer of the stereochemical information from the bond terminating in hydrogen to another bond on the chiral atom. First, the applet algorithm performs a search for *up *and *down *bonds terminated by hydrogen. If any are found, the algorithm then performs a search for another single bond to this stereocenter with minimal angle to the stereobond terminated by the hydrogen atom. This new bond replaces the hydrogen-terminated bond as the stereobond.

#### Generation of structure diagrams from double bond MCDL stereodescriptors

Similar to the atomic MCDL stereodescriptors, if the stereoconfiguration of a double bond is present in an MCDL descriptor, the specified stereoisomer is reflected in the structure diagram. In the original algorithm for structure diagram generation [2], after generation of the initial 2D atomic coordinates, bond rotations were performed to remove overlapping. In the new algorithm, prior to any bond rotation, the 2D coordinates are used to calculate MCDL double bonds stereodescriptors, which are compared with the initial ones. If the descriptors are different, a 180-degree rotation around the double bond is performed. After the correct configurations of all the stereo double bonds are established, their relative coordinates are held fixed while other bonds are allowed to rotate to remove overlap of atoms and bonds. The lack of hydrogen atoms in the structure diagrams does not cause any problems since stereoconfiguration of a double can be determined using the positions of other substituents.

#### Generation of atomic MCDL stereodescriptors from structure diagrams

**Figure 17:**
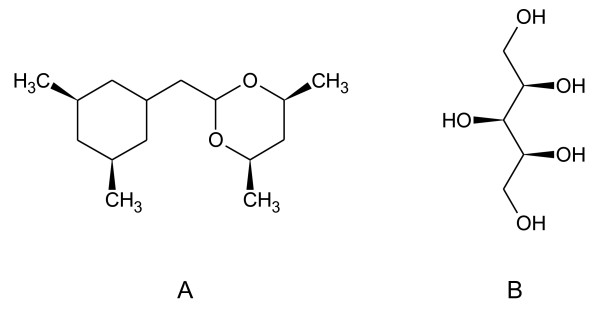
**The test structures containing multiple stereocenters**.

The Java applet algorithm employs a fast, simplified procedure for identification of stereogenic atoms. The procedure is based on hash 8-byte variables [20] for stereogenic fragments, and is designed to generate canonical descriptors except for very rare complex cases involving multiple constitutionally equivalent fragments. This is demonstrated for the two structures shown in Figure 17. For structure A, in which all the chiral centers are part of symmetrical rings, the applet gives the canonical stereodescriptor {SA:2,7,10,12,H;3,8,10,H,13;4,11,14,16,H;5,11,15,H,17}. In contrast, for the acyclic structure of B, the applet gives a valid, but non-canonical stereodescriptor {SA:1,2,3,H,6;2,1,4,H,7;3,1,5,8,H}, where the canonical descriptor is {SA:1,2,3,6,H;2,1,4,7,H;3,1,5,H,8}. Identification of the specific cases where the simplified procedures produce non-canonical stereodescriptors is a difficult task beyond the scope of this article.

#### Generation of double bond MCDL stereodescriptors from structure diagrams

To define the stereoconfiguration of a double bond, it is necessary to analyze the spatial arrangement of its four substituents (atoms or fragments). Stereodescriptors are not generated for cyclic double bonds. For acyclic double bonds, the algorithm checks for two identical terminal groups or atoms connected to either end of the double bond using atom-centered topological indexes [20]. If these indexes are the same, the stereodescriptor is not generated. If the indexes are different on both ends of the double bond and a specific configuration is present in the diagram, the algorithm generates the stereodescriptor.

### New MCDL file support in the Open Babel software package

Open Babel is a popular software package for conversion of chemical structure files from one format into others and as a C++ chemical toolkit [21]. The current version supports over 80 different chemical structure formats. Open Babel uses the SMARTS [22] language (SMILES [3] extension) for search and filtration of molecular structures. There are interfaces to other programming languages such as Perl and Python, which expand the applicability of Open Babel to other software development projects. Open Babel libraries are currently being used in more than 30 associated projects [23]. Therefore, support for the MCDL format in Open Babel provides a valuable opportunity to expand the usage of the MCDL.

Addition of the MCDL to Open Babel required the creation of new software modules. For example, chemical bond orders and atomic coordinates are not stored in many MCDL descriptors since this information is considered to be supplemental [1]. These structural parameters must be calculated during the format conversion process and molecular image generation since the majority of other chemical formats require them. Also, the existing C++ libraries of the Open Babel project did not contain modules for acyclic bond order reconstruction (*kekule.cpp *module is designed to handle aromatic bonds) and structure image generation. The required basic algorithms for bond order reconstruction and chemical structure image generation were taken from our previous effort [2] with appropriate modifications to fit the Open Babel specifications. The conversion capabilities to and from MCDL appear in Open Babel v2.3.0.

Methods for the generation of 2D coordinates derived from the Structure Editor significantly expand the utility of the Open Babel package. For example, structure image generation is now possible from other coordinate-less chemical structure formats, such as SMILES [3,4] and InChI [5]. In addition, new methods have been created (1) to check for overlapped atoms and bonds in a molecule; and, if found, to rotate the affected fragments 180 degrees around an acyclic bond or to increase the length of this acyclic bond in cases where the rotation does not work; (2) to generate the list of topologically equivalent atoms necessary to accelerate the overlapped fragment adjustment process; (3) to create the simplest image of chain structures, cycles, and condensed cycles; and (4) to calculate chiral characteristics of an atom [24].

All the new classes and methods developed for MCDL inclusion in Open Babel have been written to comply with the Open Babel documentation [25] and are compiled in a separate plugin module to facilitate their use in Open Babel applications. The LINDES [1] program code (with the minor modifications such as using object-oriented methods, bond order reconstruction, and structure diagram generation procedures) is used in this module to execute the required MCDL format support functions.

## Conclusions

### Software testing

To facilitate testing of the MCDL Java Chemical Structure Editor using large databases, we developed a standalone utility *JAmodule *that can be used to process the data in batch mode. This module allows conversion of an SDF batch file into an MCDL batch file and vice versa. The original non-stereo Java algorithm [2] was tested by performing conversions of structure files from MOLFILE format into MCDL format and back. If everything worked correctly, the final output should match the original input. The same procedure was used for testing the stereo Java algorithms presented in this paper.

The following differences were found in non-stereo testing of 20,000-records in the ChemDiv database [26]:

1. Porphyrines. Differences were found for 2 compounds from the set of 3 in the database. It was mentioned previously [2] that adequate representation of cyclooctatetraene-type structures capable of valence isomerism and valence tautomerism requires additional MCDL descriptors with information regarding specific bond order distribution in a molecule. The porphyrines belong to this class of compounds.

2. Differences for two other compounds were caused by erroneous structures. Both molecules contained atoms with illegal valences.

The structure diagram quality tests were performed using the Knovel database [27], which contains a diverse set of organic compounds with applications in many different areas. To improve the graphic performance of the Java applet, we expanded the database of pre-defined templates [2] from 105 to 145. There was no overlapping in any of the structure diagrams. Visual observation showed that more than 90% of the structure drawings were of typographic quality.

Finally, we used the public domain NSC database [28] to test the accuracy of the Java MCDL stereodescriptor algorithm. Of the 42,247 records in the database, 5,188 structures contain *up *and *down *bonds and were initially selected for testing. However, manual checks found that many of these structures did not actually contain stereo elements, i.e., structures containing *up *and *down *bonds attached to non-chiral centers. These structures were removed to yield the final data set comprised of 2,418 records.

**Figure 18:**
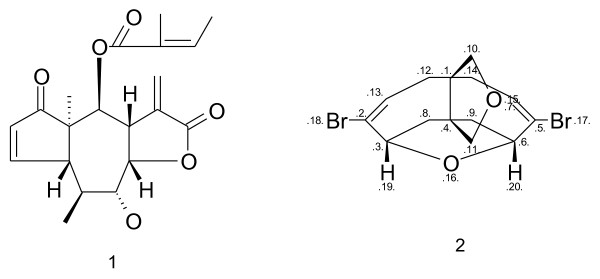
**The graphical representation of chemical structures in the NSC database **[28]**for which determinations of stereo configurations of double bonds (1) and atoms (2) were difficult to accomplish**.

After repeated conversions of MOLFILE formats into MCDL formats and back, 16 structures had differences in stereo configurations. In most of these, the major problem was the poor quality of the initial drawing. For example, an almost linear configuration of bonds around sp^2 ^atoms leads to ambiguity in determination of the *Z*/*E *configuration (Figure 18, 1). Sometimes the stereo notations *up *and *down *were used for illustrative purposes in symmetrical, non-chiral structures (Figure 18, 2). The Java algorithm recognition settings were developed with the assumption that the angles between the bonds of sp^2 ^atoms would be approximately 120 degrees, and the bond distances would be nearly equivalent. It should be noted that the comparison of initial and final structures was examined by a modified CheD program [29] that uses somewhat different algorithms of structure drawing analysis compared to the Java applet.

The latest versions of MCDL Java Chemical Structure Editor public domain source codes and executables are deposited on SourceForge [30].

## Competing interests

The authors declare that they have no competing interests.

## Authors' contributions

AAG participated in design and coordination of the study including development of MCDL concept, and edited the manuscript for publication. MNB developed MCDL concept, designed LINDES and PREPROCESS software, and took part in preparation of the manuscript. SVT and AVY developed and tested MCDL Java Chemical Structure Editor and MCDL Open Babel software, and took part in preparation of the manuscript. All authors have read and approved the final manuscript.

## Appendixes

### Appendix 1

Basic molecular topology does not represent 3D features of molecular objects, so distinctively different 3D molecular objects may have identical MCDL composition and connectivity modules (e.g., conformers and stereoisomers). Unlike conformers, stereoisomers do not undergo inter-conversion in normal conditions due to restricted internal motion. For example, the high-energy barrier of rotation around double bonds leads to "cis/trans (E/Z)" isomerism. Similarly, the high-energy barrier of inversion of four-coordinated carbon atom leads to "L/D (R/S)" isomerism. Typically, this inter-conversion barrier should be at least 20-30 Kcal/mole for stereoisomers to exist as separable compounds at room temperatures. There are many examples where these energy barriers may be lower or higher depending on environment variables (solvents, pH). In addition, compounds within the same structural group may have a wide range of inter-conversion barriers so some of them can be considered as stereoisomers, and others - as conformers (see Table one in reference [8] as an example).

### Appendix 2

An auxiliary program PREPROCESS (see additional file 3) is available to facilitate processing of MOLFILES that contain several disjointed structures. This program is designed to split a MOLFILE containing disjointed structures into a set of MOLFILES each containing only one joint structure or a fragment. These files can be used as input for LINDES 2.8 software.

## Supplementary Material

Additional file 1 **mcdl_allene**. Stereochemical modules for allenes (2 pages). Click here for file

Additional file 2 **lindes28**. The source code of the C program "LINDES" (version 2.8, 47 pages). Click here for file

Additional file 3 **preprocess**. The source code of the C program "PREPROCESS" (version 1.0, 6 pages). Click here for file
