# Supplementary material for: Modular Chemical Descriptor Language (MCDL): Stereochemical modules
Source: J Cheminform. 2011 Jan 31;3:5. doi: 10.1186/1758-2946-3-5 (PMC3042968; doi:10.1186/1758-2946-3-5)
Supplement: Additional file 1 — mcdl_allene. Stereochemical modules for allenes (2 pages). [file 1758-2946-3-5-S1.PDF]

Modular Chemical Descriptor Language (MCDL): Stereochemical Modules

Andrei A. Gakh,[1,]* Michael N. Burnett,[1] Sergei V. Trepalin,[2] Alexander V. Yarkov[2]

[1] Oak Ridge National Laboratory, Oak Ridge, Tennessee 37831, USA, e-mails AAG: gakhaa@yahoo.com; MNB: burnettmn@ornl.gov, tel. +1-865-574-1000.

[2] Institute Physiologically Active Compounds, Russian Academy of Sciences, 142432, Chernogolovka, Moscow region, Russia, e-mails SVT: trep@chemical-block.com; AVY: yarkov@ipac.ac.ru

* Author to whom correspondence should be addressed.

Compounds with an even number of cumulative double bonds (as in allene) are capable of having mirror-image stereoisomers rather than the geometrical (cis-trans) stereoisomers possible when the number of cumulative double bonds is odd. The stereochemistry of such systems is specified in the MCDL as shown in the following example.

2-Bromo-2,3-pentadiene (CH3CBr=C=CHCH3) has MCDL descriptor C;CBr;CH;2CHHH[2,3;4;5] and exists as a pair of enantiomers. Figure S1 shows the configuration of one of these isomers with the MCDL priorities of the structural fragments indicated. To specify the stereochemistry, the molecule must be reoriented with the higher priority end of the double bond system in front and the lower priority end directly behind it. The molecule must also be oriented so the substituents on the front atom lie on the vertical with the higher priority substituent up. This results in the substituents on the rear atom appearing on the horizontal. (Note: this is not a Fischer projection since the bonds represented by the vertical line are coming toward the observer and the horizontal bonds are going back. This is the opposite of a Fischer projection.)

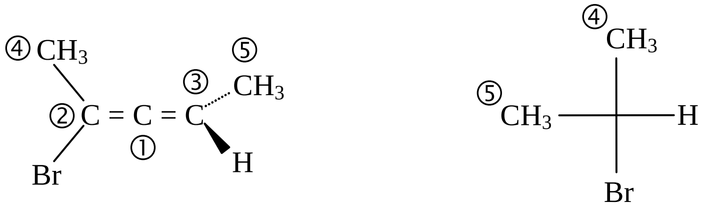

Figure S1

With this orientation, the stereochemistry is specified as {SB:2x3,4,H,Br,5}, in which 2x3 represents the fragments on the two ends of the double bond system and the four substituents are listed in clockwise order starting at the top.
