# Supplementary material for: Modular Chemical Descriptor Language (MCDL): Stereochemical modules
Source: J Cheminform. 2011 Jan 31;3:5. doi: 10.1186/1758-2946-3-5 (PMC3042968; doi:10.1186/1758-2946-3-5)
Supplement: Additional file 2 — lindes28. The source code of the C program "LINDES" (version 2.8, 47 pages). [file 1758-2946-3-5-S2.PDF]

```
/*******************************************************************/
/* lindes.c - Program to create MCDL linear descriptor             */
/* Version 2.8 July 2007                                           */
/* Mike Burnett, Oak Ridge National Laboratory                     */
/*                                                                 */
/* Disclaimer of Liability                                         */
/*    This software was prepared as an account of work sponsored   */
/*    by an agency of the U.S. Government. Neither the U.S.        */
/*    Government nor any agency thereof, or any of their employees, */
/*    makes any warranty, express or implied, or assumes any legal  */
/*    liability or responsibility for the accuracy, completeness,   */
/*    or usefulness of any information contained herein or results  */
/*    produced from application of this software, or represents that */
/*    its use would not infringe privately owned rights.           */
/*******************************************************************/

#include <stdio.h>
#include <stdlib.h>
#include <math.h>

#define TRUE    1
#define FALSE   0

#define MOL        0
#define FRAGCON    1

#define MAXFRAGS   100
#define MAXBONDS   400
#define MAXCHARS   1000

// Flags
#define VERBOSE    TRUE
#define MOLOUTPUT  TRUE
#define WEB        FALSE

// Double bond carbon end types
#define TWOFRAGS   0
#define FRAGATOM   1
#define TWOATOMS   2

typedef int Int4[4];
typedef double Dbl3[3];
typedef char Chr12[12];
typedef int Int3[3];
typedef char FileLine[81];

// MOL file information
FileLine *molfline;
int molflinecount, molfatoms, molfbonds;

struct stereoatom
{
       int sf[3];
       char atom[12];
};

struct stereoatomset
```

```
{
       int molatom, fischerposition, flag;
       char symbol[3];
       double x,y,z;
};

typedef struct stereoatomset SAS[5];

FILE *o_verbose;
int  inputtype, MOLatom[MAXFRAGS], maxdepth;
void exitprog(int);
int freeflag;

main(int argc, char **argv)
{
    FILE *i_file, *o_file;
    char a, filename[82];
    void MOL2FragCon(), addMOL2ld(FILE *), FragCon2ld(FILE *, FILE *);
    void ReadMolFile(char *), CheckMOLFile();

    freeflag = 0;
    inputtype = MOL;

    if (argc == 2) // argument on command line assumes MOL input
        strcpy(filename,argv[1]);
    else // get user input
    {
        printf("LinDes 2.8 (July 2007)\n\n");
        printf("Input MOL file name: ");
        scanf("%s",filename);
    }

    if (VERBOSE)
    {
        o_verbose = fopen("details.txt","w");
        fprintf(o_verbose,"Input file: %s\n",filename);
    }

    if (inputtype == MOL)
    {
        ReadMolFile(filename);              // read and save MOL file
        CheckMOLFile();                     // check MOL input for
substructures
        MOL2FragCon();                      // convert MOL input to FRAGCON
        i_file = fopen("lindes.inp","r");
    }
    else
        i_file = fopen(filename,"r");

    if (i_file == NULL)
    {
        printf("Invalid file name.\n");
        exitprog(freeflag);
    }

    o_file = fopen("lindes.txt","w");
```

```
    /* input now in FRAGCON format - create linear descriptor */
    FragCon2ld(i_file, o_file);

    /* if MOL input, add supplementary modules to descriptor */
    if (inputtype == MOL && MOLOUTPUT)
        addMOL2ld(o_file);

    fclose(o_file);

    if (VERBOSE)
       fclose(o_verbose);

    /* needed for MCDL web site only */
    if (WEB)
    {
        i_file = fopen("lindes.txt","r");
        o_file = fopen("lindes1.txt","w");
        a = fgetc(i_file);
        while (a != EOF)
        {
            if (a != '\n')
                fputc(a,o_file);
            a = fgetc(i_file);
        }
        fputc('\n',o_file);
        fflush(o_file);
        fclose(o_file);
        fclose(i_file);
        i_file = fopen("lindes1.txt","r");
        o_file = fopen("lindes2.txt","w");
        a = fgetc(i_file);
        while (a != '{' && a != EOF)
        {
            fputc(a,o_file);
            a = fgetc(i_file);
        }
        fputc('\n',o_file);
        fflush(o_file);
        fclose(o_file);
        fclose(i_file);
        o_file = fopen("success.txt","w");
        fprintf(o_file,"1\n");
        fflush(o_file);
        fclose(o_file);
    }

    exitprog(freeflag);
}

// Read and save original MOL file in global variable molfline
void ReadMolFile(char *molfilename)
{
     FILE *i_file;
     FileLine line;
     int linecount;

     if ((i_file = fopen(molfilename,"r")) == NULL)
```

```
     {
        printf("Bad input file.\n");
        exitprog(freeflag);
     }

     molflinecount = 0;
     while (fgets(line,81,i_file))
           molflinecount++;

     molfline = (FileLine *) malloc(molflinecount*sizeof(FileLine));

     freeflag = 1;

     rewind(i_file);

     for (linecount=0; linecount<molflinecount; linecount++)
     {
         fgets(molfline[linecount],81,i_file);
         molfline[linecount][strlen(molfline[linecount])-1] = '\0';
     }

     fclose(i_file);

     strncpy(line,molfline[3],3);
     sscanf(line,"%d",&molfatoms);
     strncpy(line,&molfline[3][3],3);
     sscanf(line,"%d",&molfbonds);
}

/* Check MOL file for unconnected substructures */
void CheckMOLFile()
{
    int *atoms, i, found;
    Int4 *bonds;
    char line[81];

    atoms = (int *) malloc(molfatoms*sizeof(int));
    bonds = (Int4 *) malloc(molfbonds*sizeof(Int4));

    for (i=0; i<molfatoms; i++)
        atoms[i] = 0;

    // read bond block
    for (i=0; i<molfbonds; i++)
    {
        strcpy(line,molfline[4+molfatoms+i]);
        sscanf(line,"%d %d",&bonds[i][0],&bonds[i][1]);
        bonds[i][2] = 0;
    }

    // beginning with first pair of bonded atoms,
    // iteratively find all atoms connected to these
    atoms[bonds[0][0]-1] = 1;
    atoms[bonds[0][1]-1] = 1;
    bonds[0][2] = 1;
    found = 1;
    while (found)
```

```
    {
        found = 0;
        for (i=1; i<molfbonds; i++)
            if (!bonds[i][2])
               if (atoms[bonds[i][0]-1] == 0 && atoms[bonds[i][1]-1] == 0)
                  continue;
               else
               {
                  atoms[bonds[i][0]-1] = 1;
                  atoms[bonds[i][1]-1] = 1;
                  bonds[i][2] = 1;
                  found++;
               }
    }

    // check atom list for any unconnected atoms
    for (i=0; i<molfatoms; i++)
        if (atoms[i] == 0)
        {
           printf("Structure contains unconnected substructures.
Exiting.\n");
           free(atoms);
           free(bonds);
           exitprog(freeflag);
        }

    free(atoms);
    free(bonds);
}

/* convert MOL file input to FRAGCON format */
void MOL2FragCon()
{
    FILE *i_file,*o_file;
    int  i,j,k,m,n,q,nbonds,nfrags,fragnum,nb,nf,fp,nb_save,numcaps;
    int  charge,nstereo,scenter,sc,flag3d,symbolnums[4];
    char line[81],temp[4],fg[10],symbol[4][3];
    void doMOLCHG(char *, Int4 *, int *);
    void arrangefragment(char *, char *);
    Chr12 *fragment, *tfr;
    Int4 *ia, *tcon;
    Dbl3 *coord;
    int  *chg, *h, tatom(char *), *chiralcandidate;
    SAS *sas;
    void coords2fischer(struct stereoatomset *);
    //int stereobond(struct stereoatomset *,int,int,int *,int *,int *);
    int stereobond(SAS *,int,int,int *,int *,int *,int);
    void ordersymbols(int, char [][3],int *);
    int findchiralcandidates(int, Chr12 *, int, Int4 *, int, int *);
    int cleanconnections(int, int, Int4 *, Chr12 *);

    nfrags = molfatoms;
    nbonds = molfbonds;

    fragment = (Chr12 *) malloc(nfrags*sizeof(Chr12));
    coord = (Dbl3 *) malloc(nfrags*sizeof(Dbl3));
    chg = (int *) malloc(nfrags*sizeof(int));
```

```
h = (int *) malloc(nfrags*sizeof(int));
ia = (Int4 *) malloc(nbonds*sizeof(Int4));
chiralcandidate = (int *) malloc(nfrags*sizeof(int));

// read atom block
flag3d = FALSE;
for (i=0; i<nfrags; i++)
{
    strcpy(line,molfline[4+i]);
    strcat(line," 0 0");
    sscanf(line,"%lf %lf %lf %s %*d %d",&coord[i][0],&coord[i][1],
                     &coord[i][2],fragment[i],&charge);
    if (charge == 0)
        chg[i] = 0;
    else
        chg[i] = 4 - charge;
    MOLatom[i] = i+1;
    if (coord[i][2] > .01)
       flag3d = TRUE;
}

// read bond block
for (i=0; i<nbonds; i++)
{
    strcpy(line,molfline[4+molfatoms+i]);
    strcat(line," 0 0");
    /* read bond: from, to, type, updown */
    sscanf(line,"%d %d %d %d",&ia[i][0],&ia[i][1],&ia[i][2],&ia[i][3]);
    if (ia[i][2] > 3)
    {
        printf("Bond type %d not yet implemented.\n",
            ia[i][2]);
        exitprog(freeflag);
    }
}

nb_save = nbonds;

// Look for and process possible chiral centers
for (i=0; i<nfrags; i++)
    chiralcandidate[i] = 0;
nstereo = findchiralcandidates(flag3d, fragment, nfrags, ia, nbonds,
                                       chiralcandidate);

sas = (SAS *) malloc(nstereo*sizeof(SAS));

if (nstereo)
{
   m = -1;
   for (n=0; n<nfrags; n++)
   {
       if (chiralcandidate[n] == 0)
          continue;
       m++;
       scenter = n+1;
       sas[m][0].molatom = scenter;
       strcpy(sas[m][0].symbol,fragment[scenter-1]);
```

```
            sas[m][0].x = coord[scenter-1][0];
            sas[m][0].y = coord[scenter-1][1];
            sas[m][0].z = coord[scenter-1][2];
            sas[m][0].fischerposition = 0;
            //printf("Stereocenter: %d
%s\n",sas[m][0].molatom,sas[m][0].symbol);

//printf("%10.4f,%10.4f,%10.4f\n",sas[m][0].x,sas[m][0].y,sas[m][0].z);
            j = 1;
            for (i=0; i<nbonds; i++)
                if ((ia[i][0] == scenter) || (ia[i][1] == scenter))
                {
                   if (ia[i][0] == scenter)
                      k = ia[i][1] - 1;
                   else
                      k = ia[i][0] - 1;
                   sas[m][j].molatom = k+1;
                   strcpy(sas[m][j].symbol,fragment[k]);
                   sas[m][j].x = coord[k][0];
                   sas[m][j].y = coord[k][1];
                   sas[m][j].z = coord[k][2];
                   if (ia[i][3] == 1)
                      sas[m][j].z = 1.;
                   if (ia[i][3] == 6)
                      sas[m][j].z = -1.;
                   //printf("%d %s\n",sas[m][j].molatom,sas[m][j].symbol);
                   //printf("%10.4f,%10.4f,%10.4f\n",sas[m][j].x,sas[m][j].y,
                              //sas[m][j].z);
                   j++;
                }
            coords2fischer(sas[m]);
            //for (i=0; i<4; i++)
                //printf("%d\n",sas[m][i+1].fischerposition);
        }
    }

    // determine number of Hs per atom and add to fragment string --
C,O,N,P,S ONLY!!
    for (i=0; i<nfrags; i++)
    {
        fragnum = i+1;
        h[i] = 0;
        k = 0;
        for (j=0; j<nb_save; j++)
            if (ia[j][0] == fragnum || ia[j][1] == fragnum)
                k += ia[j][2];

        if (strcmp(fragment[i],"C") == 0)
        {
            if ((chg[i] == -1 || chg[i] == 1) && k < 3)     // carbon with
charge
               h[i] = 3-k;
            if (chg[i] == 0)
               h[i] = 4-k;
        }
        else if (strcmp(fragment[i],"O") == 0)
        {
```

```
        if (chg[i] == -1 && k < 1)     // oxygen with negative charge
           h[i] = 1-k;
        if (chg[i] == 1 && k < 3)      // oxygen with positive charge
           h[i] = 3-k;
        if (chg[i] == 0)
           h[i] = 2-k;
    }
    else if (strcmp(fragment[i],"N") == 0)
    {
        if (chg[i] == 1 && k < 4)      // nitrogen with positive charge
           h[i] = 4-k;
        if (chg[i] == 0)
           h[i] = 3-k;
    }
    else if (strcmp(fragment[i],"P") == 0)
        h[i] = 3-k;
    else if (strcmp(fragment[i],"S") == 0)
        h[i] = 2-k;

    if (h[i] < 0)
        h[i] = 0;

    for (j=0; j<h[i]; j++)
        strcat(fragment[i],"H");
}

nbonds = nb_save;
nf = nfrags;
i = 0;
while (i < nfrags)
{
    k = 0;
    fragnum = i+1;
    for (j=0; j<nbonds; j++)
        if (ia[j][0] == fragnum || ia[j][1] == fragnum)
            k++;

    // lindes 2.8 correction for descriptor errors resulting
    // from order of atoms in atom list in molfile
    // no fragment with two non-'H' uppercase letters can combine
    // with another fragment
    numcaps = 0;
    m = strlen(fragment[i]);
    for (j=0; j<m; j++)
        if (isupper(fragment[i][j]) && (fragment[i][j] != 'H'))
           numcaps++;

    if (k == 1 && h[i] == 0 && numcaps < 2)
    {
        for (j=0; j<nbonds; j++)
        {
            if (ia[j][0] == fragnum)
            {
                strcat(fragment[ia[j][1]-1],fragment[i]);
                chg[ia[j][1]-1] += chg[i];
                break;
            }
```

```
            if (ia[j][1] == fragnum)
            {
                strcat(fragment[ia[j][0]-1],fragment[i]);
                chg[ia[j][0]-1] += chg[i];
                break;
            }
        }
        if (fragnum < nfrags)
        {
            for (j=i; j<nfrags-1; j++)
            {
                strcpy(fragment[j],fragment[j+1]);
                chg[j] = chg[j+1];
                h[j] = h[j+1];
                MOLatom[j] = MOLatom[j+1];
            }
            i--;
        }
        nf--;
        nb = nbonds;
        for (j=0; j<nbonds; j++)
            if (ia[j][0] == fragnum || ia[j][1] == fragnum)
            {
                for (k=j; k<nbonds-1; k++)
                {
                    ia[k][0] = ia[k+1][0];
                    ia[k][1] = ia[k+1][1];
                    ia[k][2] = ia[k+1][2];
                }
                nb--;
            }
        nbonds = nb;
        for (j=0; j<nbonds; j++)
        {
            if (ia[j][0] > fragnum)
                ia[j][0]--;
            if (ia[j][1] > fragnum)
                ia[j][1]--;
        }
    }
    i++;
}

nfrags = nf;

o_file = fopen("lindes.inp","w");

// Fragments: number, symbol, charge
for (i=0; i<nfrags; i++)
{
    arrangefragment(fragment[i],fg);
    fprintf(o_file,"%3d %s %d\n",i+1,fg,chg[i]);
    strcpy(fragment[i],fg);
}

tfr = (Chr12 *) malloc((nbonds+nstereo*4)*sizeof(Chr12));
tcon = (Int4 *) malloc((nbonds+nstereo*4)*sizeof(Int4));
```

```
q = -1;

for (m = 0; m < nstereo; m++)
    for (i=1; i<5; i++)
        sas[m][i].flag = FALSE;

// Connections: from, to, type, fischerposition, [atomic symbol]
for (i = 0; i < nbonds; i++)
{
    q++;
    if (nstereo && stereobond(sas,ia[i][0],ia[i][1],&nf,&nb,&fp,nstereo))
    {
          tcon[q][0] = nf;
          tcon[q][1] = nb;
          tcon[q][2] = ia[i][2];
          tcon[q][3] = fp;
          strcpy(tfr[q],fragment[tcon[q][1]-1]);
    }
    else
    {
       tcon[q][0] = ia[i][0];
       tcon[q][1] = ia[i][1];
       tcon[q][2] = ia[i][2];
       tcon[q][3] = 0;
       strcpy(tfr[q],fragment[tcon[q][1]-1]);
    }
}

// Add any missing stereoatom information
if (nstereo)
   for (i = 0; i < nbonds; i++)
       if (stereobond(sas,ia[i][0],ia[i][1],&nf,&nb,&fp,nstereo))
       {
          q++;
          tcon[q][0] = nf;
          tcon[q][1] = nb;
          tcon[q][2] = ia[i][2];
          tcon[q][3] = fp;
          strcpy(tfr[q],fragment[tcon[q][1]-1]);
       }


// Terminal atom connections
for (m = 0; m < nstereo; m++)
{
   k = 0;
   for (i=1; i<5; i++)
       if (tatom(sas[m][i].symbol))
          strcpy(symbol[k++],sas[m][i].symbol);

   if (k > 0)
   {
      ordersymbols(k,symbol,symbolnums);

      for (i=0; i<nfrags; i++)
          if (MOLatom[i] == sas[m][0].molatom)
          {
```

```
                sc = i+1;
                break;
             }

         for (i=1; i<5; i++)
             if (tatom(sas[m][i].symbol))
             {
                for (j=0; j<k; j++)
                    if (strcmp(sas[m][i].symbol,symbol[j]) == 0)
                    {
                       nb = symbolnums[j];
                       break;
                    }
                q++;
                tcon[q][0] = sc;
                tcon[q][1] = nb;
                tcon[q][2] = 1;
                tcon[q][3] = sas[m][i].fischerposition;
                strcpy(tfr[q],sas[m][i].symbol);
             }
      }
   }

   q++;
   if (VERBOSE)
   {
      fprintf(o_verbose, "Initial connection table:\n");
      for (m = 0; m < q; m++)
          fprintf(o_verbose, "%3d %-4d %d %d %s\n", tcon[m][0], tcon[m][1],
                                 tcon[m][2], tcon[m][3], tfr[m]);
      fprintf(o_verbose, "Final connection table:\n");
   }
   q = cleanconnections(q, nfrags, tcon, tfr);
   for (m = 0; m < q; m++)
   {
       fprintf(o_file,"%3d %-4d %d %d %s\n", tcon[m][0], tcon[m][1],
                               tcon[m][2], tcon[m][3], tfr[m]);
       if (VERBOSE)
          fprintf(o_verbose,"%3d %-4d %d %d %s\n", tcon[m][0], tcon[m][1],
                               tcon[m][2], tcon[m][3], tfr[m]);
   }

   fclose(o_file);

   free(fragment);
   free(chg);
   free(h);
   free(ia);
   free(coord);
   free(chiralcandidate);
   free(sas);
   free(tcon);
   free(tfr);
}

// arrange terminal atoms of structural fragment in
// alphabetical order following nonterminal atom
```

```
void arrangefragment(char *a, char *fg)
{
    char ntatom[3], tatom[4][3], q[24];
    int  start, numterminal, strindex, i, j;

    // extract non-terminal atom from fragment string
    ntatom[0] = a[0];
    if (islower(a[1]))
    {
        ntatom[1] = a[1];
        ntatom[2] = '\0';
        start = 2;
    }
    else
    {
        ntatom[1] = '\0';
        start = 1;
    }

    // extract terminal atoms, if any, from fragment string
    strindex = 0;
    numterminal = 0;
    for (i = start; i < strlen(a); i++)
    {
        if (isupper(a[i]))
        {
            q[strindex++] = ' ';
            numterminal++;
        }
        q[strindex++] = a[i];
    }
    q[strindex] = '\0';
    if (numterminal == 0 || numterminal == 1)
    {
        strcpy(fg,a);
        return;
    }
    strcat(q," a b c d");
    sscanf(q,"%s %s %s %s",tatom[0],tatom[1],tatom[2],tatom[3]);

    /* sort terminal atoms in ASCII order */
    for (i = 0; i < numterminal-1; i++)
        for (j = i; j < numterminal; j++)
            if (strcmp(tatom[i],tatom[j]) > 0)
            {
                strcpy(q,tatom[i]);
                strcpy(tatom[i],tatom[j]);
                strcpy(tatom[j],q);
            }

    /* build sorted fragment string */
    strcpy(q,ntatom);
    for (i = 0; i < numterminal; i++)
        strcat(q,tatom[i]);

    strcpy(fg,q);
}
```

```
/* interpret charge information from MOL input */
void doMOLCHG(char *a, Int4 *ka, int *nb)
{
    int  numcharges,atom[8],charge[8],i,j;
    char z[200];

    sscanf(a,"%*s %*s %d", &numcharges);

    strcpy(z,a);
    strcat(z," 9 9 9 9 9 9 9 9 9 9 9 9 9 9 9 9");
    sscanf(z,"%*s %*s %*d %d %d %d %d %d %d %d %d %d %d %d %d %d %d %d %d",
            &atom[0], &charge[0], &atom[1], &charge[1],
            &atom[2], &charge[2], &atom[3], &charge[3],
            &atom[4], &charge[4], &atom[5], &charge[5],
            &atom[6], &charge[6], &atom[7], &charge[7]);

    for (j=0; j<numcharges; j++)
    {
        ka[*nb][0] = atom[j];
        ka[*nb][1] = 0;
        ka[*nb][2] = -charge[j];
        (*nb)++;
    }
}

/* add MOL info as supplementary modules to linear descriptor */
void addMOL2ld(FILE *o_f)
{
    int   i, from, to, type;
    int   zvals, count;
    float x, y, z;
    char  line[82], symbol[3], dummy[12];
    static char bondtype[8] = {'s','d','t','a','r','v','z','n'};

    fprintf(o_f,"{Z1:%s}\n", molfline[0]);
    fprintf(o_f,"{Z2:%s}\n", molfline[1]);
    fprintf(o_f,"{Z3:%s}\n", molfline[2]);
    fprintf(o_f,"{NA:%d}", molfatoms);
    fprintf(o_f,"{NB:%d}", molfbonds);

    zvals = 0;
    for (i=0; i<molfatoms; i++)
    {
        sscanf(molfline[4+i],"%f %f %f", &x, &y, &z);
        if (z > 0.0001)
        {
           zvals = 1;
           break;
        }
    }

    if (zvals)
        fprintf(o_f,"{ZV:Y}\n");
    else
        fprintf(o_f,"{ZV:N}\n");
```

```
fprintf(o_f,"{CC:");

count = 0;
for (i=0; i<molfatoms; i++)
{
    if (i > 0)
        fprintf(o_f,";");
    if (zvals == 0 && count == 5)
    {
        fprintf(o_f,"\n");
        count = 0;
    }
    else if (zvals == 1 && count == 3)
    {
        fprintf(o_f,"\n");
        count = 0;
    }
    strcpy(line,molfline[i+4]);
    strcat(line, "  0  0");
    sscanf(line,"%f %f %f %s", &x, &y, &z, symbol);

    //x += minx;
    //y += miny;
    //z += minz;
    sprintf(dummy,"%.5f",x);
    sscanf(dummy,"%f",&x);
    sprintf(dummy,"%.5f",y);
    sscanf(dummy,"%f",&y);
    fprintf(o_f,"%g,%g", x, y);
    count++;
    if (zvals)
    {
        sprintf(dummy,"%.5f",z);
        sscanf(dummy,"%f",&z);
        fprintf(o_f,",%g", z);
    }
    fprintf(o_f,"%s", symbol);
}

fprintf(o_f,"}\n{BB:");

count = 0;
for (i=0; i<molfbonds; i++)
{
    if (i > 0)
        fprintf(o_f,";");
    if (count == 8)
    {
        fprintf(o_f,"\n");
        count = 0;
    }
    sscanf(molfline[i+4+molfatoms],"%d %d %d", &from, &to, &type);
    fprintf(o_f,"%d%c%d", from, bondtype[type-1], to);
    count++;
}

fprintf(o_f,"}");
```

```
    count = 4 + molfatoms + molfbonds;

    for (i=count; i<molflinecount; i++)
    {
        if (molfline[i][0] == 'M' && strncmp(&molfline[i][3],"END",3) != 0)
        {
            strcpy(line,&molfline[i][3]);
            fprintf(o_f,"\n{MM:%s}",line);
        }
    }

    fprintf(o_f,"\n");
}

int  nbonds,ntatoms;
char finalstr[MAXCHARS];
int  kflag=0;

/* create linear descriptor from FRAGCON input */
void FragCon2ld(FILE *i_file, FILE *o_file)
{
    int  i,j,k,n,nn,nt,ntypes,charge,first;
    int  ltypes,ia0,ia1,chgflag,fischer,nsbonds,db;
    int  icons[6],chg[MAXFRAGS],nchg[MAXFRAGS];
    char constr[MAXCHARS];
    char semis[100];
    int  comma;
    void solve(int, Int4 *, Int4 *, int *, int);
    char *stereoorder(struct stereoatom *, char *);
    char *stereodb(int, int, Int3 *, char *);
    FILE *uniq_file;

    int nconnections, nsatoms, ndbonds;
    int newone, netcharge;   //flags
    Chr12 *fragment, *fs;
    char frag[12], line[82];
    int *ifragnum, *ix, *qx;
    Int4 *ia, *qa;
    struct stereoatom *sa, sax[4];
    Int3 *map;
    int *ca;

    // count fragments
    // ntatoms: total number of fragments
    // nconnections: total number of connections in file
    // nsatoms: number of stereoatoms
    // ndbonds: number of double bonds
    ntatoms = 0;
    nsatoms = 0;
    ndbonds = 0;
    while(fgets(line,81,i_file))
    {
        sscanf(line,"%*d %s",frag);
        if (!isalpha(frag[0]))
           break;
        ntatoms++;
```

```
}
line[strlen(line)-1] = '\0';
strcat(line," 0 0 0");
sscanf(line,"%*d %*d %d %d",&db,&fischer);
if (fischer == 1)
   nsatoms++;
if (db == 2)
   ndbonds++;
nconnections = 1;
while(fgets(line,81,i_file))
{
    nconnections++;
    line[strlen(line)-1] = '\0';
    strcat(line," 0 0 0");
    sscanf(line,"%*d %*d %d %d",&db,&fischer);
    if (fischer == 1)
       nsatoms++;
    if (db == 2)
       ndbonds++;
}

rewind(i_file);

// reread fragments and find different types
// ntypes: number of fragment types
// fragment[i]: atomic symbol string of fragment type i
// netcharge: net charge of structure
ntypes = 0;
chgflag = FALSE;
netcharge = 0;
fragment = (Chr12 *) malloc(ntatoms*sizeof(Chr12));
fs       = (Chr12 *) malloc(ntatoms*sizeof(Chr12));
ca       = (int *) malloc(ntatoms*sizeof(int));
for (j = 0; j < ntatoms; j++)
{
    fgets(line,81,i_file);
    // add 0 charge if none present
    line[strlen(line)-1] = '\0';
    strcat(line," 0");
    sscanf(line,"%d %s %d",&n,frag,&charge);
    ca[j] = charge;
    netcharge += charge;
    if (charge != 0)
       chgflag = TRUE;
    newone = TRUE;
    for (i = 0; i < ntypes; i++)
        if (strcmp(fragment[i],frag) == 0)
        {
            newone = FALSE;
            break;
        }
    if (newone)
        strcpy(fragment[ntypes++],frag);
}

ltypes = ntypes;   // ??
```

```
// order fragment types in ASCII order
for (i = 0; i < ntypes-1; i++)
    for (j = i+1; j < ntypes; j++)
        if (strcmp(fragment[j],fragment[i]) < 0)
        {
            strcpy(frag,fragment[i]);
            strcpy(fragment[i],fragment[j]);
            strcpy(fragment[j],frag);
        }

// ifragnum[i]: number of fragments of type i
// ix[i]: fragment type of fragment i
ix = (int *) malloc(ntatoms*sizeof(int));
ifragnum = (int *) malloc(ntypes*sizeof(int));
for (i=0; i<ntypes; i++)
    ifragnum[i] = 0;

// reread the fragments
rewind(i_file);
for (i = 0; i < ntatoms; i++)
{
    fgets(line,81,i_file);
    sscanf(line,"%*d %s",fs[i]);
    for (j = 0; j < ntypes; j++)
        if (strcmp(fs[i],fragment[j]) == 0)
        {
            ix[i] = j+1;
            ifragnum[j]++;
        }
}

// read in the connections
// ia[i][0]: from fragment; ia[i][1]: to fragment of connection i
ia = (Int4 *) malloc(2*nconnections*sizeof(Int4));
sa = (struct stereoatom *) malloc(4*nsatoms*sizeof(struct stereoatom));
nbonds = 0;
nsbonds = 0;
for (i = 0; i < nconnections; i++)
{
    fgets(line,81,i_file);
    // add 0 charge if none present    ???
    line[strlen(line)-1] = '\0';
    strcat(line," 0 0 0");
    sscanf(line,"%d %d %*d %d %s",&ia0,&ia1,&fischer,constr);
    // Save bond information for atomic stereochemistry
    if (fischer)
    {
       sa[nsbonds].sf[0] = ia0;
       sa[nsbonds].sf[1] = ia1;
       sa[nsbonds].sf[2] = fischer;
       if (ia1 > 9000)
          strcpy(sa[nsbonds++].atom,constr);
       else
          strcpy(sa[nsbonds++].atom,fs[ia1-1]);
    }

    newone = TRUE;
```

```
        for (j = 0; j < nbonds; j++)
            if ((ia[j][0] == ia0 && ia[j][1] == ia1) ||
                (ia[j][0] == ia1 && ia[j][1] == ia0))
            {
                newone = FALSE;
                break;
            }
        if (newone)
        {
            if (ia1 < 9000)
            {
               ia[nbonds][0] = ia0;
               ia[nbonds++][1] = ia1;
               ia[nbonds][0] = ia1;
               ia[nbonds++][1] = ia0;
            }
        }
    }

    fclose(i_file);
    free(fs);

    for (i = 0; i < nbonds; i++)
    {
        ia[i][2] = ix[ia[i][0]-1];
        ia[i][3] = ix[ia[i][1]-1];
    }

    qa = (Int4 *) malloc(nbonds*sizeof(Int4));
    qx = (int *) malloc(ntatoms*sizeof(int));
    maxdepth = 1;

    if (VERBOSE)
        fprintf(o_verbose,"Candidate Connectivity Strings\n");

    /* data ready for solution */
    solve(ntypes,ia,qa,qx,1);

    free(ix);
    ix = qx;
    free(ia);
    ia = qa;

    if (ntatoms == 1)      // !!!!!!!!!!!!!!!!!!!!!!!!!!!!!!!!!!!
       ix[0] = 1;

/* Output */

    /* original and final fragment numbers */
    if (VERBOSE)
    {
        fprintf(o_verbose,"Max. recursion level: %d\n",maxdepth);
        fprintf(o_verbose,"Fragment Numbers\nOriginal   Final\n");
        for (i=0; i<ntatoms; i++)
            if (inputtype == FRAGCON)
                fprintf(o_verbose,"%5d      %3d\n",i+1,ix[i]);
                //fprintf(o_verbose,"  -   %5d      %3d\n",i+1,ix[i]);
```

```
            else
                fprintf(o_verbose,"%5d      %3d\n", MOLatom[i],ix[i]);
                //fprintf(o_verbose,"%5d   %5d   %3d\n",
MOLatom[i],i+1,ix[i]);
    }

    if (chgflag)
        for (i=0; i<ntatoms; i++)
            nchg[ix[i]] = chg[i];

    /* net charge */
    if (netcharge != 0)
    {
        if (netcharge > 0)
            sprintf(line,"%d+;",netcharge);
        else
            sprintf(line,"%d-;",abs(netcharge));
        fprintf(o_file,"%s",line);
        if (VERBOSE)
            fprintf(o_verbose,"%s",line);
    }

    /* fragment portion of linear descriptor */
    constr[0] = '\0';
    for (i = 0; i < ltypes; i++)
    {
        if (i > 0)
            strcat(constr,";");
        if (ifragnum[i] > 1)
        {
            sprintf(line,"%d",ifragnum[i]);
            strcat(constr,line);
        }
        sprintf(line,"%s",fragment[i]);
        strcat(constr,line);
    }
    fprintf(o_file,"%s\n",constr);

    uniq_file = fopen("unique.txt","w");
    fprintf(uniq_file,"%s",constr);

    if (VERBOSE)
        fprintf(o_verbose,"%s\n",constr);


    if (VERBOSE)
        fprintf(o_verbose,"\nInitial Connectivity\n[");
    sprintf(constr,"[");

    /* connectivity portion of linear descriptor */
    semis[0] = '\0';
    nt = 1;
    for (i=0; i<ntatoms; i++)
    {
        if (i > 0)
        {
            if (VERBOSE)
```

```
            fprintf(o_verbose,";");
        strcat(semis,";");
    }

    if (VERBOSE && nt%10 == 0)
        fprintf(o_verbose,"\n");
    n = 0;
    for (j=0; j<nbonds; j++)
        if (ia[j][2] == (i+1))
            icons[n++] = ia[j][3];
    if (n > 1)
    {
        for (j=0; j<n-1; j++)
            for (k=j+1; k<n; k++)
                if (icons[k] < icons[j])
                {
                    nn = icons[j];
                    icons[j] = icons[k];
                    icons[k] = nn;
                }

    }
    comma = 0;
    for (j=0; j<n; j++)
    {
        if (j > 0 && VERBOSE)
            fprintf(o_verbose,",");
        if (VERBOSE)
            fprintf(o_verbose,"%d",icons[j]);
        if (icons[j] > (i+1) && comma == 0)
        {
            sprintf(line,"%s",semis);
            strcat(constr,line);
            sprintf(line,"%d",icons[j]);
            strcat(constr,line);
            comma = 1;
            semis[0] = '\0';
        }
        else if (icons[j] > (i+1) && comma == 1)
        {
            sprintf(line,",%d",icons[j]);
            strcat(constr,line);
        }

    }
    nt++;
}

sprintf(line,"]\n");
strcat(constr,line);

fprintf(o_file,"%s", constr);

fprintf(uniq_file,"%s\n",constr);
fclose(uniq_file);

if (VERBOSE)
```

```
{
    fprintf(o_verbose,"]\nTrimmed Connectivity\n");
    fprintf(o_verbose,"%s", constr);
}

/* Stereo atoms */
if (nsatoms)
{
   if (VERBOSE)
      fprintf(o_verbose,"Stereo Atoms\n");
   fprintf(o_file,"{SA:");

   newone = FALSE;

   for (i = 0; i < nsatoms*4; i++)
   {
       sa[i].sf[0] = ix[sa[i].sf[0]-1];
       if (sa[i].sf[1] < 9000)
          sa[i].sf[1] = ix[sa[i].sf[1]-1];
   }

   for (i = 0; i < ntatoms; i++)
   {
       n = 0;
       for (j = 0; j < nsatoms*4; j++)
       {
           if (sa[j].sf[0] != (i+1))
              continue;
           sax[n].sf[0] = sa[j].sf[0];
           sax[n].sf[1] = sa[j].sf[1];
           sax[n].sf[2] = sa[j].sf[2];
           strcpy(sax[n++].atom,sa[j].atom);
           if (VERBOSE)
              fprintf(o_verbose,"%d %d %d %s\n",sax[n-1].sf[0],
                      sax[n-1].sf[1],sax[n-1].sf[2],sax[n-1].atom);
       }
       if (n == 4)
       {
          if (VERBOSE)
             fprintf(o_verbose,"%s\n",stereoorder(sax,constr));
          if (newone)
             fprintf(o_file,";");
          fprintf(o_file,"%s",stereoorder(sax,constr));
          newone = TRUE;
       }
    }
    fprintf(o_file,"}\n");
}

/* Stereo double bonds */
if (ndbonds && (inputtype == MOL))
{
    map = (Int3 *) malloc(ntatoms*sizeof(Int3));
    for (i=0; i<ntatoms; i++)
    {
        map[i][0] = MOLatom[i];
        map[i][1] = i+1;
```

```
            map[i][2] = ix[i];
        }
        strcpy(constr,stereodb(ndbonds,nconnections,map,constr));
        if (strcmp(constr,"X") != 0)
        {
           fprintf(o_file,"%s", constr);
           if (VERBOSE)
              fprintf(o_verbose,"%s", constr);
        }
        free(map);
    }

    /* fragment charges */
    if (chgflag)
    {
        strcpy(constr,"{CZ:");
        first = TRUE;
        for (i=1; i<=ntatoms; i++)
            for (j=0; j<ntatoms; j++)
                if (ix[j] == i && ca[j])
                {
                   if (first)
                   {
                      if (ca[j] == 1)
                         sprintf(line,"%d,1+",ix[j]);
                      else
                         sprintf(line,"%d,1-",ix[j]);
                      first = FALSE;
                   }
                   else
                      if (ca[j] == 1)
                         sprintf(line,";%d,1+",ix[j]);
                      else
                         sprintf(line,";%d,1-",ix[j]);
                   strcat(constr,line);
                   break;
                }
        strcat(constr,"}\n");
        fprintf(o_file,"%s", constr);

        if (VERBOSE)
        {
            fprintf(o_verbose,"Fragment Charges\n");
            fprintf(o_verbose,"%s", constr);
        }
    }

    free(fragment);
    free(ix);
    free(ifragnum);
    free(ia);
}

/* solves for linear descriptor -- may call itself */
void solve(int ntypes, Int4 *z, Int4 *qa, int *qx, int depth)
{
    int  i,dupnum,iflag,j,k,newone,ktype,nn,nt;
```

```
int  nsum[MAXBONDS][MAXFRAGS],lflag[MAXFRAGS];
char strg[MAXFRAGS+1],strngs[MAXFRAGS][MAXFRAGS+1];
char tstr[MAXFRAGS+1];
char *constring(int z[][4], char *);
int  numdups, dupfrag, jump, jflag;
int  ix[MAXFRAGS],conntab[MAXBONDS][4],cx[MAXFRAGS];
int  mx[MAXFRAGS];

/* depth = recursion level */
if (depth > 10)
{
    printf("Ten recursion levels exceeded.\n");
    exitprog(freeflag);
}
if (depth > maxdepth)
    maxdepth = depth;

for (i=0; i<nbonds; i++)
{
    for (j=0; j<4; j++)
        conntab[i][j] = z[i][j];
    mx[conntab[i][0]-1] = conntab[i][2];
}

jflag = TRUE;
if (depth == 1)
{
    jflag = FALSE;
    for (i=0; i<ntatoms; i++)
    {
        ix[i] = mx[i];
        if (ix[i] != mx[0])
            jflag = TRUE;
    }
}

iflag = TRUE;
while (iflag)
{
    if (jflag)
        for (i=0; i<ntatoms; i++)
            ix[i] = 0;

        nt = 1;
        ktype = 1;
        iflag = FALSE;

       while (nt <= ntypes && jflag)
       {
        for (j=0; j<ntatoms; j++)
        {
               lflag[j] = FALSE;
            for (i=0; i<nbonds; i++)
                   nsum[i][j] = 0;
        }

        for (i=0; i<nbonds; i++)
```

```
    if (conntab[i][2] == nt)
    {
        j = conntab[i][0]-1;
        k = conntab[i][3]-1;
        nsum[j][k]++;
        lflag[j] = TRUE;
    }

        nn = 0;

    for (i=0; i<ntatoms; i++)
    if (lflag[i])
    {
        for (j=0; j<ntypes; j++)
            strg[j] = nsum[i][j]+48;
        strg[ntypes] = '\0';
        newone = TRUE;
        for (j=0; j<nn; j++)
            if (strcmp(strngs[j],strg) == 0)
            {    newone = FALSE;
                break;
            }
        if (newone)
            strcpy(strngs[nn++],strg);
    }

    if (nn > 1)
    {
    iflag = TRUE;

    /* sort strings */
    for (i=0; i<nn-1; i++)
        for (j=i+1; j<nn; j++)
            if (strcmp(strngs[j],strngs[i]) > 0)
            {
            strcpy(tstr,strngs[i]);
            strcpy(strngs[i],strngs[j]);
            strcpy(strngs[j],tstr);
            }
        }

    for (i=0; i<ntatoms; i++)
    if (lflag[i])
    {
        for (j=0; j<ntypes; j++)
            strg[j] = nsum[i][j]+48;
        strg[ntypes] = '\0';
        for (j=0; j<nn; j++)
            if (strcmp(strg,strngs[j]) == 0)
                ix[i] = ktype+j;
    }

        nt++;
        ktype += nn;
   }

ntypes = 0;
```

```
for (i=0; i<nbonds; i++)
{
    conntab[i][2] = ix[conntab[i][0]-1];
    conntab[i][3] = ix[conntab[i][1]-1];
    if (conntab[i][2] > ntypes)
        ntypes = conntab[i][2];
}

    if (iflag)
    continue;

if (ntypes < ntatoms)
{
    for (j=0; j<ntypes; j++)
    {
    numdups = 0;
    for (i=0; i<ntatoms; i++)
        if (ix[i] == j+1)
        {
            numdups++;
            jump = ix[i] - mx[i];
        }
    if (numdups > 1)
    {
        dupfrag = j+1;
        break;
    }
    }

    for (i=0; i<ntatoms; i++)
        cx[i] = ix[i];

    for (j=0; j<numdups; j++)
    {
        for (i=0; i<ntatoms; i++)
            ix[i] = cx[i];

    dupnum = 0;
        for (i=0; i<ntatoms; i++)
    {
        if (ix[i] > dupfrag)
            ix[i] = mx[i] + jump + 1;
        else if (ix[i] == dupfrag && j != dupnum)
        {
            dupnum++;
            ix[i] = mx[i] + jump + 1;
        }
        else if (ix[i] == dupfrag && j == dupnum)
            dupnum++;
    }

    ntypes = 0;
        for (i=0; i<nbonds; i++)
        {
        conntab[i][2] = ix[conntab[i][0]-1];
        conntab[i][3] = ix[conntab[i][1]-1];
        if (conntab[i][2] > ntypes)
```

```
                ntypes = conntab[i][2];
            }
            solve(ntypes,conntab,qa,qx,depth+1);
        }
    }
}

if (ntypes == ntatoms)
{
        if (VERBOSE)
        fprintf(o_verbose,"%s\n",constring(conntab,tstr));

    if (!kflag)
    {
        kflag++;
        strcpy(finalstr,constring(conntab,tstr));
        for (i=0; i<ntatoms; i++)
            qx[i] = ix[i];
        for (i=0; i<nbonds; i++)
            for (j=0; j<4; j++)
                qa[i][j] = conntab[i][j];
    }
    else if (strcmp(finalstr,constring(conntab,tstr)) != 0)
    {
        for (i=0; i<ntatoms; i++)
        {
            tstr[0] = '\0';
            strg[0] = '\0';
            for (j=0; j<ntatoms; j++)
            {
                strcat(tstr,"0");
                strcat(strg,"0");
            }
            for (j=0; j<nbonds; j++)
            {
                if (conntab[j][2] == (i+1))
                       tstr[conntab[j][3]-1] = '1';
                if (qa[j][2] == (i+1))
                       strg[qa[j][3]-1] = '1';
            }
            if (strcmp(tstr,strg) < 0)
                   break;
            if (strcmp(tstr,strg) > 0)
            {
                strcpy(finalstr,constring(conntab,tstr));
                for (j=0; j<ntatoms; j++)
                    qx[j] = ix[j];
                for (j=0; j<nbonds; j++)
                    for (k=0; k<4; k++)
                        qa[j][k] = conntab[j][k];
                break;
            }
        }
    }
}
}
```

```
/* Create descriptor connectivity string from connectivity table of fragments
*/
char *constring(int conntab[][4], char *tstr)
{
    int  i,j,k,n,nn,icons[6],comma;
    char line[82],semis[100];
    char *sp = tstr;

    sprintf(sp,"[");
    semis[0] = '\0';

    for (i=0; i<ntatoms; i++)
    {
        if (i > 0)
            strcat(semis,";");

        n = 0;
        for (j=0; j<nbonds; j++)
            if (conntab[j][2] == (i+1))
                icons[n++] = conntab[j][3];
        if (n > 1)
        {
            for (j=0; j<n-1; j++)
                for (k=j+1; k<n; k++)
                    if (icons[k] < icons[j])
                    {
                        nn = icons[j];
                        icons[j] = icons[k];
                        icons[k] = nn;
                    }

        }
        comma = 0;
        for (j=0; j<n; j++)
        {
            if (icons[j] > (i+1) && comma == 0)
            {
                sprintf(line,"%s%d",semis,icons[j]);
                strcat(sp,line);
                comma = 1;
                semis[0] = '\0';
            }
            else if (icons[j] > (i+1) && comma == 1)
            {
                sprintf(line,",%d",icons[j]);
                strcat(sp,line);
            }

        }
    }

    strcat(sp,"]");
    return(sp);
}

// Create stereo atom module string for descriptor
char *stereoorder(struct stereoatom *sax, char *tstr)
```

```
{
        char *sp = tstr, a[5];
        int i, j, fr[4], temp, ndex, switches, center;

        center = sax[0].sf[0];

        // Order fragments up, down, left, right
        for (i = 0; i < 4; i++)
            for (j = 0; j < 4; j++)
                if (sax[j].sf[2] == i+1)
                   fr[i] = sax[j].sf[1];

        switches = 0;

        // Bring two highest priority fragments to up and down postions
        for (i = 0; i < 2; i++)
        {
            temp = 10000;
            ndex = i;
            for (j = i; j < 4; j++)
                if (fr[j] < temp)
                {
                   temp = fr[j];
                   ndex = j;
                }

            if (ndex > i)
            {
               temp = fr[i];
               fr[i] = fr[ndex];
               fr[ndex] = temp;
               switches++;
            }
        }

        // If only one exchange has occurred, exchange left and right
        if (switches%2)
        {
           temp = fr[2];
           fr[2] = fr[3];
           fr[3] = temp;
        }

        sprintf(sp,"%d,",center);
        for (i = 0; i < 4; i++)
        {
            if (fr[i] < 9000) // fragment connection
            {
               sprintf(a,"%d",fr[i]);
               strcat(sp,a);
            }
            else              // atom connection
               for (j = 0; j < 4; j++)
                   if (sax[j].sf[1] == fr[i])
                   {
                      strcat(sp,sax[j].atom);
                      break;
```

```
                    }
                if (i < 3)
                   strcat(sp,",");
            }

            return(sp);
}

char *stereodb(int ndbonds, int nconnections, Int3 *map, char *tstr)
{
     FILE *i_file;
     int  i, j, k, n, ppath[4], hswitch[2], ifrag[4], conjugated;
     int  path[4], pri, first, endtype[2], *flag, start, sameside;
     char frag[4][12], line[82], afragment[12], atom0[12], atom3[12];
     char *sp = tstr, sym[4][3];
     Int4 *ia;
     Chr12 *fragment;
     int *connflag, firstdbatom, seconddbatom, thirdsubst, fourthsubst;
     struct dbe {int fragnum, partner, conn_num[2];char conn_frag[2][12];}
dbend[2];
     int samesideofdb(int *, char *, char *);
     int highprisub(int,int,char *,char *,char *, int *);
     int dbinring(int, int, Int4 *, int *, int);
     int dbendisok(char *, int *, char *, char *);
     int dbendfragsok(int, int, Int4 *, Chr12 *, char [][12], int *, int,
int);

     ia = (Int4 *) malloc(nconnections*sizeof(Int4));
     fragment = (Chr12 *) malloc((ntatoms+1)*sizeof(Chr12));
     flag = (int *) malloc(nconnections*sizeof(int));
     connflag = (int *) malloc(nconnections*sizeof(int));

     for (i = 0; i < nconnections; i++)
         flag[i] = FALSE;

     // Get fragments and connections from fragcon file
     i_file = fopen("lindes.inp","r");
     for (i = 0; i < ntatoms; i++)
     {
        fgets(line,81,i_file);
        sscanf(line,"%*d %s",afragment);
        for (j = 0; j < ntatoms; j++)
            if ((i+1) == map[j][1])
            {
               strcpy(fragment[map[j][2]],afragment);
               break;
            }
     }
     for (i = 0; i < nconnections; i++)
     {
        fgets(line,81,i_file);
        sscanf(line,"%d %d %d",&ia[i][0],&ia[i][1],&ia[i][2]);
        for (j = 0; j < ntatoms; j++)
            if (ia[i][0] == map[j][1])
            {
               ia[i][0] = map[j][2];
               break;
```

```
            }
        if (ia[i][1] < 9000)
           for (j = 0; j < ntatoms; j++)
              if (ia[i][1] == map[j][1])
              {
                 ia[i][1] = map[j][2];
                 break;
              }
      }
      fclose(i_file);

      start = TRUE;
      for (i = 1; i <= ntatoms; i++)
          for (j = 0; j < nconnections; j++)
          {
              if (ia[j][2] != 2)
                 continue;
              if (flag[j])
                 continue;
              if ((ia[j][0] != i) && (ia[j][1] != i))
                 continue;
              if ((fragment[ia[j][0]][0] != 'C') || (fragment[ia[j][1]][0] !=
'C'))
              {
                 if (VERBOSE)
                    fprintf(o_verbose,"Double bond %d=%d is not carbon-
carbon.\n",
                            ia[j][0],ia[j][1]);
                 continue;
              }
              for (k = 0; k < nconnections; k++)
                  connflag[k] = FALSE;
              connflag[j] = TRUE;
              if (dbinring(ia[j][0],ia[j][1],ia,connflag,nconnections))
              {
                 if (VERBOSE)
                    fprintf(o_verbose,"Double bond %d=%d in ring.\n",
                            ia[j][0],ia[j][1]);
                 continue;
              }
              flag[j] = TRUE;
              if (!dbendisok(fragment[ia[j][0]],&endtype[0],sym[0],sym[1]))
              {
                 if (VERBOSE)
                    fprintf(o_verbose,"Atom %d of double bond %d=%d has two
identical atoms attached.\n",
                            ia[j][0],ia[j][0],ia[j][1]);
                 continue;
              }
              if (!dbendisok(fragment[ia[j][1]],&endtype[1],sym[2],sym[3]))
              {
                 if (VERBOSE)
                    fprintf(o_verbose,"Atom %d of double bond %d=%d has two
identical atoms attached.\n",
                            ia[j][1],ia[j][0],ia[j][1]);
                 continue;
              }
```

```
             for (k = 0; k < 4; k++)
                 ifrag[k] = 0;
             if ((endtype[0] == TWOFRAGS) || (endtype[0] == FRAGATOM))
                if (!dbendfragsok(ia[j][0], ia[j][1], ia, fragment,
                      frag, ifrag, 0, nconnections))
                {
                   if (VERBOSE)
                      fprintf(o_verbose,"Atom %d of double bond %d=%d has two
identical fragments attached.\n",
                           ia[j][0],ia[j][0],ia[j][1]);
                   continue;
                }
             if ((endtype[1] == TWOFRAGS) || (endtype[1] == FRAGATOM))
                if (!dbendfragsok(ia[j][1], ia[j][0], ia, fragment,
                      frag, ifrag, 1, nconnections))
                {
                   if (VERBOSE)
                      fprintf(o_verbose,"Atom %d of double bond %d=%d has two
identical fragments attached.\n",
                           ia[j][1],ia[j][0],ia[j][1]);
                   continue;
                }
             /*conjugated = 0;
             for (k = 0; k < 4; k++)
             {
                 for (n = 0; n < nconnections; n++)
                     if (((ia[n][0] == ifrag[k]) && (ia[n][2] == 2)) ||
                         ((ia[n][1] == ifrag[k]) && (ia[n][2] == 2)))
                     {
                         conjugated = 1;
                         break;
                     }
                 if (conjugated)
                    break;
             }
             if (conjugated)
             {
                if (VERBOSE)
                   fprintf(o_verbose,"Conjugated double bond found.\n");
                continue;
             }   */
             for (k = 0; k < 2; k++)
             {
                 dbend[k].fragnum = ia[j][k];
                 dbend[k].partner = ia[j][(k+1)%2];

                 if (endtype[k] == TWOFRAGS)
                 {
                    dbend[k].conn_num[0] = ifrag[2*k];
                    strcpy(dbend[k].conn_frag[0],frag[2*k]);
                    dbend[k].conn_num[1] = ifrag[2*k+1];
                    strcpy(dbend[k].conn_frag[1],frag[2*k+1]);
                    continue;
                 }
                 if (endtype[k] == FRAGATOM)
                 {
                    dbend[k].conn_num[0] = 0;
```

```
               strcpy(dbend[k].conn_frag[0],sym[2*k]);
               dbend[k].conn_num[1] = ifrag[2*k];
               strcpy(dbend[k].conn_frag[1],frag[2*k]);
               continue;
            }
            if (endtype[k] == TWOATOMS)
            {
               dbend[k].conn_num[0] = 0;
               strcpy(dbend[k].conn_frag[0],sym[2*k]);
               dbend[k].conn_num[1] = 0;
               strcpy(dbend[k].conn_frag[1],sym[2*k+1]);
               continue;
            }
         }

         if (VERBOSE)
         {
            fprintf(o_verbose,"Stereo double bond\n");
            for (k = 0; k < 2; k++)
            {
               fprintf(o_verbose,"C:%d\n",dbend[k].fragnum);
               fprintf(o_verbose,"Substituent:%d
%s\n",dbend[k].conn_num[0],
                       dbend[k].conn_frag[0]);
               fprintf(o_verbose,"Substituent:%d
%s\n",dbend[k].conn_num[1],
                       dbend[k].conn_frag[1]);
            }
         }

         strcpy(atom0,"X");
         strcpy(atom3,"X");

         if (dbend[0].fragnum < dbend[0].partner)
         {
            path[1] = dbend[0].fragnum;
            path[2] = dbend[0].partner;
            first = 0;
         }
         else
         {
            path[1] = dbend[0].partner;
            path[2] = dbend[0].fragnum;
            first = 1;
         }

         for (k = 0; k < 2; k++)
         {
             hswitch[k] = FALSE;
             pri = highprisub(dbend[k].conn_num[0],dbend[k].conn_num[1],

dbend[k].conn_frag[0],dbend[k].conn_frag[1],afragment,
                      &hswitch[k]);
             if (k == first)
             {
               path[0] = pri;
               if (path[0] == 0)
```

```
            strcpy(atom0,afragment);
      }
      else
      {
         path[3] = pri;
         if (path[3] == 0)
            strcpy(atom3,afragment);
      }
}

for (k = 0; k < 4; k++)
    for (n = 0; n < ntatoms; n++)
        if (path[k] == map[n][2])
        {
           path[k] = map[n][0];
           break;
        }

sameside = samesideofdb(path,atom0,atom3);

for (k = 0; k < 4; k++)
{
    ppath[k] = 0;
    for (n = 0; n < ntatoms; n++)
        if (path[k] == map[n][0])
        {
           ppath[k] = map[n][2];
           break;
        }
}

if (start)
   sprintf(sp,"{SB:");
else
   strcat(sp,";");
start = FALSE;
sprintf(line,"%dd%d,",ppath[1],ppath[2]);
strcat(sp,line);
for (k = 0; k < 2; k++)
    if (dbend[k].fragnum == ppath[1])
    {
       firstdbatom = k;
       seconddbatom = (k+1)%2;
    }
// First substituent of double bond
if (hswitch[0])
{
   sprintf(line,"H,");
   if (strcmp(dbend[firstdbatom].conn_frag[0],"H") == 0)
      fourthsubst = 1;
   else
      fourthsubst = 0;
}
else
   if (path[0] > 0)
   {
      sprintf(line,"%d,",ppath[0]);
```

```
                if (dbend[firstdbatom].conn_num[0] == ppath[0])
                   fourthsubst = 1;
                else
                   fourthsubst = 0;
             }
             else
             {
                sprintf(line,"%s,",atom0);
                if (strcmp(dbend[firstdbatom].conn_frag[0],atom0) == 0)
                   fourthsubst = 1;
                else
                   fourthsubst = 0;
             }
          strcat(sp,line);
          // Second substituent of double bond
          if (sameside)
             if (ppath[3] > 0)
             {
                sprintf(line,"%d",ppath[3]);
                if (dbend[seconddbatom].conn_num[0] == ppath[3])
                   thirdsubst = 1;
                else
                   thirdsubst = 0;
             }
             else
                if (hswitch[1])
                {
                   sprintf(line,"H");
                   if (strcmp(dbend[seconddbatom].conn_frag[0],"H") == 0)
                      thirdsubst = 1;
                   else
                      thirdsubst = 0;
                }
                else
                {
                   sprintf(line,"%s",atom3);
                   if (strcmp(dbend[seconddbatom].conn_frag[0],atom3) ==
0)

                      thirdsubst = 1;
                   else
                      thirdsubst = 0;
                }
          else
          {
             first = (first+3)%2;
             if (dbend[first].conn_num[0] != ppath[3])
                if (dbend[first].conn_num[0] > 0)
                {
                   sprintf(line,"%d",dbend[first].conn_num[0]);
                   thirdsubst = 1;
                }
                else
                   if (hswitch[1])
                   {
                      sprintf(line,"H");
                      if (strcmp(dbend[seconddbatom].conn_frag[0],"H") ==
0)
```

```
                                thirdsubst = 1;
                             else
                                thirdsubst = 0;
                          }
                          else
                          {
                             sprintf(line,"%s",dbend[first].conn_frag[0]);
                             thirdsubst = 1;
                          }
                    else
                       if (strcmp(dbend[first].conn_frag[0],atom3) == 0)
                          if (hswitch[1])
                          {
                             sprintf(line,"H");
                             if (strcmp(dbend[seconddbatom].conn_frag[0],"H") ==
0)

                                thirdsubst = 1;
                             else
                                thirdsubst = 0;
                          }
                          else
                          {
                             sprintf(line,"%s",dbend[first].conn_frag[1]);
                             thirdsubst = 0;
                          }
                       else
                          if (hswitch[1])
                          {
                             sprintf(line,"H");
                             if (strcmp(dbend[seconddbatom].conn_frag[0],"H") ==
0)

                                thirdsubst = 1;
                             else
                                thirdsubst = 0;
                          }
                          else
                          {
                             sprintf(line,"%s",dbend[first].conn_frag[0]);
                             thirdsubst = 1;
                          }
                 }
                 strcat(sp,line);
                 // Third substituent of double bond
                 if (dbend[seconddbatom].conn_num[thirdsubst] > 0)
                    sprintf(line,",%d",dbend[seconddbatom].conn_num[thirdsubst]);
                 else

sprintf(line,",%s",dbend[seconddbatom].conn_frag[thirdsubst]);
                 strcat(sp,line);
                 // Fourth substituent of double bond
                 if (dbend[firstdbatom].conn_num[fourthsubst] > 0)
                    sprintf(line,",%d",dbend[firstdbatom].conn_num[fourthsubst]);
                 else

sprintf(line,",%s",dbend[firstdbatom].conn_frag[fourthsubst]);
                 strcat(sp,line);
             }
```

```
     if (!start)
        strcat(sp,"}");

     free(ia);
     free(fragment);
     free(flag);
     free(connflag);

     if (start)
        strcpy(sp,"X");
     return sp;
}

// Return TRUE if double bond is in a ring
int dbinring(int nextconn, int target, Int4 *ia, int *connflag, int nc)
{
    int i, j;

    for (i = 0; i < nc; i++)
    {
        if (connflag[i] == TRUE)
           continue;
        for (j = 0; j < 2; j++)
        {
            if (ia[i][j] == nextconn)
            {
               connflag[i] = TRUE;
               if (ia[i][(j+1)%2] == target)
                  return TRUE;
               if (dbinring(ia[i][(j+1)%2],target,ia,connflag,nc))
                  return TRUE;
            }
        }
    }
    return FALSE;
}

int dbendisok(char *a, int *endtype, char *sym1, char *sym2)
{
    int i, numcaps, lastcap;

    if (strlen(a) == 1)                // 2 fragments
    {
       *endtype = TWOFRAGS;
       return TRUE;
    }
    numcaps = 0;
    for (i = 1; i < strlen(a); i++)
        if (isupper(a[i]))
           numcaps++;
    if (numcaps == 1)                  // 1 fragment, 1 atom
    {
       strcpy(sym1,&a[1]);
       *endtype = FRAGATOM;
       return TRUE;
    }
```

```
    for (i = strlen(a); i > 0; i--)
        if (isupper(a[i-1]))
        {
           lastcap = i-1;
           break;
        }
    strcpy(sym1,&a[lastcap]);
    a[lastcap] = '\0';
    strcpy(sym2,&a[1]);
    if (strcmp(sym1,sym2) == 0)        // 2 identical atoms
       return FALSE;

    *endtype = TWOATOMS;               // 2 different atoms
    return TRUE;
}

int dbendfragsok(int from, int to, Int4 *ia, Chr12 *frag,
                      char fr[][12], int *ifr, int call, int nc)
{
    int i, n;

    n = 0;
    for (i = 0; i < nc; i++)
    {
        if ((ia[i][0] == from) && (ia[i][1] != to))
        {
           ifr[2*call+n] = ia[i][1];
           strcpy(fr[2*call+n],frag[ia[i][1]]);
           n++;
        }
        if ((ia[i][1] == from) && (ia[i][0] != to))
        {
           ifr[2*call+n] = ia[i][0];
           strcpy(fr[2*call+n],frag[ia[i][0]]);
           n++;
        }
    }
    if ((n == 2) && (strcmp(fr[2*call],fr[2*call+1]) == 0))
       return FALSE;

    return TRUE;
}

// Returns TRUE if two double bond substituents are on same side of d.b.
int samesideofdb(int *path, char *atom0, char *atom3)
{
     char line[82];
     int i, j, numatoms, numbonds, count, atomflag, tpath[4], fr, to, flag3d;
     Chr12 *fragment;
     double delx, dely, delz, bondlength, angle, c1, c2;
     struct {double x,y,z;} point[4];

     flag3d = FALSE;
     atomflag = FALSE;
     for (i = 0; i < 4; i++)
     {
         tpath[i] = path[i];
```

```
          if (tpath[i] == 0)
             atomflag = TRUE;
      }

      numatoms = molfatoms;
      numbonds = molfbonds;

      if (atomflag)
      {
         fragment = (Chr12 *) malloc(numatoms*sizeof(Chr12));
         for (i = 0; i < numatoms; i++)
             sscanf(molfline[4+i],"%*f %*f %*f %s",fragment[i]);
         for (i = 0; i < numbonds; i++)
         {
             sscanf(molfline[4+molfatoms+i],"%d %d",&fr,&to);
             if (((fr == tpath[1]) && (to == tpath[2])) ||
                 ((to == tpath[1]) && (fr == tpath[2])))
                continue;
             if (tpath[0] == 0)
                if ((to == tpath[1]) && (strcmp(atom0,fragment[fr-1]) == 0))
                   tpath[0] = fr;
                else if ((fr == tpath[1]) && (strcmp(atom0,fragment[to-1]) ==
0))
                   tpath[0] = to;
             if (tpath[3] == 0)
                if ((to == tpath[2]) && (strcmp(atom3,fragment[fr-1]) == 0))
                   tpath[3] = fr;
                else if ((fr == tpath[2]) && (strcmp(atom3,fragment[to-1]) ==
0))
                   tpath[3] = to;
         }

         free(fragment);
      }

      count = 0;
      for (i = 0; i < numatoms; i++)
      {
          strcpy(line,molfline[4+i]);
          for (j = 0; j < 4; j++)
              if (tpath[j] == i+1)
              {
                 sscanf(line,"%lf %lf %lf", &point[j].x, &point[j].y,
&point[j].z);
                 if (point[j].z > .01)
                    flag3d = TRUE;
                 count++;
                 break;
              }
          if (count == 4)
             break;
      }

      // Move point 1 to origin
      delx = point[1].x;
      dely = point[1].y;
      delz = point[1].z;
```

```
for (i = 0; i < 4; i++)
{
    point[i].x -= delx;
    point[i].y -= dely;
    point[i].z -= delz;
}

if (flag3d)
{
   // Rotate around Y to bring point 2 into XY plane
   bondlength = sqrt(point[2].x*point[2].x + point[2].z*point[2].z);
   angle = asin(-point[2].z/bondlength);
   if (point[2].x < 0.)
      angle = -angle;

   for (i = 0; i < 4; i++)
   {
       c1 = point[i].x * cos(angle) - point[i].z * sin(angle);
       c2 = point[i].x * sin(angle) + point[i].z * cos(angle);
       point[i].x = c1;
       point[i].z = c2;
   }
}

// Rotate around Z to bring point 2 along Y
bondlength = sqrt(point[2].x*point[2].x + point[2].y*point[2].y);
angle = asin(-point[2].x/bondlength);
if (point[2].y < 0.)
   angle = -angle;

for (i = 0; i < 4; i++)
{
    c1 = point[i].x * cos(angle) + point[i].y * sin(angle);
    c2 = point[i].x * -sin(angle) + point[i].y * cos(angle);
    point[i].x = c1;
    point[i].y = c2;
}

if (flag3d)
{
   // Rotate around Y to bring point 0 into XY plane
   bondlength = sqrt(point[0].x*point[0].x + point[0].z*point[0].z);
   angle = asin(-point[0].z/bondlength);
   if (point[0].x < 0.)
      angle = -angle;

   for (i = 0; i < 4; i++)
   {
       c1 = point[i].x * cos(angle) - point[i].z * sin(angle);
       c2 = point[i].x * sin(angle) + point[i].z * cos(angle);
       point[i].x = c1;
       point[i].z = c2;
   }
}

if (((point[0].x > 0) && (point[3].x > 0)) ||
    ((point[0].x < 0) && (point[3].x < 0)))
```

```
        return TRUE;
     else
        return FALSE;
}

/*
int getpriorityof(int fragnum, Int3 *map)
{
    int i;

    for (i=0; i<ntatoms; i++)
        if (fragnum == map[i][1])
           return map[i][2];

    return 0;
}
*/

// Return higher priority substituent (other than H) on C of stereo double
bond
int highprisub(int pri0,int pri1,char *a1,char *a2,char *aa, int *fl)
{
     int fragnum;
     char atom[12];

     if ((pri0 > 0) && (pri1 > 0))     // two fragments
        if (pri0 < pri1)
           fragnum = pri0;
        else
           fragnum = pri1;
     else if (pri0 > 0)                // fragment and atom
        fragnum = pri0;
     else if (pri1 > 0)                // atom and fragment
        fragnum = pri1;
     else                              // two atoms
        if (strcmp(a1,a2) < 0)
        {
           fragnum = 0;
           strcpy(aa,a1);
        }
        else
        {
           fragnum = 0;
           strcpy(aa,a2);
        }

     if (strcmp(aa,"H") == 0)          // disallow H
     {
        *fl = TRUE;
        if (strcmp(a1,"H") == 0)
           strcpy(aa,a2);
        else
           strcpy(aa,a1);
     }

     return fragnum;
}
```

```
// Orient stereocenter and 4 connected atoms into Fischer projection
// and return Fischer positions of 4 connected atoms
void coords2fischer(struct stereoatomset *a)
{
    /*
    (1) Rotate molecule around Y to put 1 atom (atom1) in XY plane (x,y,0)
    (2) Rotate molecule around Z to put that same atom (atom1) along +Y
(0,+y,0)
    (3) Rotate molecule around Y to place bottom atom (atom2) in YZ plane
with
        -z (0,y,-z)
    (4) In Fischer projection, atom1 is up, atom2 is down.  Of other two
atoms,
        one with -x is left and one with +x is right.
    */

    int    i, top, bottom;
    double angle, newc1, newc2, bondlength;
    struct {double x,y,z;} point[4];

    for (i = 0; i < 4; i++)
    {
        point[i].x = a[i+1].x;
        point[i].y = a[i+1].y;
        point[i].z = a[i+1].z;
    }

    // Translate atoms to place stereocenter at origin
    for (i = 0; i < 4; i++)
    {
        point[i].x -= a[0].x;
        point[i].y -= a[0].y;
        point[i].z -= a[0].z;
    }

    fprintf(o_verbose,"Rotating around stereocenter for Fischer
projection.\n");
    for (i = 0; i < 4; i++)

fprintf(o_verbose,"%10.4f,%10.4f,%10.4f\n",point[i].x,point[i].y,point[i].z);

    top = 99;
    for (i = 0; i < 4; i++)
        if (point[i].z == 0.)
        {
           top = i;
           break;
        }

    if (top == 99)
    {
        top = 0;
        bondlength = sqrt(point[top].x*point[top].x +
                          point[top].z*point[top].z);
        angle = asin(-point[top].z/bondlength);
        if (point[top].x < 0.)
```

```
           angle = -angle;

        fprintf(o_verbose,"Y Rotation Angle = %f\n",angle*180./3.14159);

        for (i = 0; i < 4; i++)
        {
            newc1 = point[i].x * cos(angle) - point[i].z * sin(angle);
            newc2 = point[i].x * sin(angle) + point[i].z * cos(angle);
            point[i].x = newc1;
            point[i].z = newc2;

fprintf(o_verbose,"%10.4f,%10.4f,%10.4f\n",point[i].x,point[i].y,point[i].z);
        }
    }

    bondlength = sqrt(point[top].x*point[top].x +
                      point[top].y*point[top].y);
    angle = -acos(point[top].y/bondlength);
    if (point[top].x < 0.)
       angle = -angle;

    fprintf(o_verbose,"Z Rotation Angle = %f\n",angle*180./3.14159);

    for (i = 0; i < 4; i++)
    {
        newc1 = point[i].x * cos(angle) + point[i].y * sin(angle);
        newc2 = point[i].x * -sin(angle) + point[i].y * cos(angle);
        point[i].x = newc1;
        point[i].y = newc2;

fprintf(o_verbose,"%10.4f,%10.4f,%10.4f\n",point[i].x,point[i].y,point[i].z);
    }

    bottom = 99;

    for (i = 0; i < 4; i++)
    {
        if (i == top)
           continue;
        if (point[i].z < 0.)
        {
           bottom = i;
           break;
        }
    }

    bondlength = sqrt(point[bottom].x*point[bottom].x +
                      point[bottom].z*point[bottom].z);
    angle = -acos(-point[bottom].z/bondlength);
    if (point[bottom].x < 0.)
       angle = -angle;

    fprintf(o_verbose,"Y Rotation Angle = %f\n",angle*180./3.14159);

    for (i = 0; i < 4; i++)
    {
        newc1 = point[i].x * cos(angle) - point[i].z * sin(angle);
```

```
        newc2 = point[i].x * sin(angle) + point[i].z * cos(angle);
        point[i].x = newc1;
        point[i].z = newc2;

fprintf(o_verbose,"%10.4f,%10.4f,%10.4f\n",point[i].x,point[i].y,point[i].z);
    }

    a[top+1].fischerposition = 1;
    a[bottom+1].fischerposition = 2;

    for (i = 0; i < 4; i++)
    {
        if ((i == top) || (i == bottom))
           continue;
        if (point[i].x < 0.)
           a[i+1].fischerposition = 3;
        else
           a[i+1].fischerposition = 4;
    }
}

// Returns TRUE if atomic symbol is one of H, F, Cl, Br, I
int tatom(char *a)
{
    if (strcmp(a,"H") == 0)
       return TRUE;
    if (strcmp(a,"F") == 0)
       return TRUE;
    if (strcmp(a,"Cl") == 0)
       return TRUE;
    if (strcmp(a,"Br") == 0)
       return TRUE;
    if (strcmp(a,"I") == 0)
       return TRUE;

    return FALSE;
}

// Orders terminal atoms on stereocenter in ASCII order
void ordersymbols(int k, char a[][3], int *b)
{
     int i , j;
     char x[4][3], temp[3];

     if (k == 1)
     {
        b[0] = 9001;
        return;
     }

     for (i = 0; i < k; i++)
         strcpy(x[i],a[i]);

     for (i = 0; i < k-1; i++)
        for (j = i; j < k; j++)
            if (strcmp(x[i],x[j]) > 0)
            {
```

```
                strcpy(temp,x[i]);
                strcpy(x[i],x[j]);
                strcpy(x[j],temp);
            }

     for (i = 0; i < k; i++)
        for (j = 0; j < k; j++)
            if (strcmp(a[i],x[j]) == 0)
            {
               b[i] = 9001 + j;
               break;
            }
}

// Returns TRUE if connection (bond) involves a stereocenter
//int stereobond(struct stereoatomset *a,int from,int to, int *newfrom, int
*newto, int *fp)
int stereobond(SAS *a,int from,int to, int *newfrom, int *newto, int *fp, int
nstereo)
{
    int i, m;

    for (m = 0; m < nstereo; m++)
        for (i = 1; i < 5; i++)
        {
            if ((a[m][i].molatom == MOLatom[from-1]) &&
                (a[m][0].molatom == MOLatom[to-1]) && !a[m][i].flag)
            {
               *newfrom = to;
               *newto = from;
               *fp = a[m][i].fischerposition;
               a[m][i].flag = TRUE;
               return TRUE;
            }
            if ((a[m][i].molatom == MOLatom[to-1]) &&
                (a[m][0].molatom == MOLatom[from-1]) && !a[m][i].flag)
            {
               *newfrom = from;
               *newto = to;
               *fp = a[m][i].fischerposition;
               a[m][i].flag = TRUE;
               return TRUE;
            }
        }
    return FALSE;
}

int findchiralcandidates(int flag3d, Chr12 *fragment, int nfrags,
                              Int4 *ia, int nbonds, int *cc)
{
     int i, j, k, numconns, updown, nstereo;
     char atom[4][3];
     int dupatoms(char [4][3]);

     nstereo = 0;
     if (!flag3d)
     {
```

```
        updown = 0;
        for (i = 0; i < nbonds; i++)
            if (ia[i][3] > 0)
               updown++;
        if (updown == 0)
           return 0;
     }

     for (i = 1; i <= nfrags; i++)
     {
         if (strcmp(fragment[i-1],"C") != 0)
            continue;
         k = 0;
         numconns = 0;
         updown = 0;
         for (j = 0; j < nbonds; j++)
         {
             if (ia[j][0] == i)
             {
                if (ia[j][2] > 1)
                break;
                numconns++;
                strcpy(atom[k++],fragment[ia[j][1]-1]);
             }
             if (ia[j][1] == i)
             {
                if (ia[j][2] > 1)
                break;
                numconns++;
                strcpy(atom[k++],fragment[ia[j][0]-1]);
             }
             if (((ia[j][0] == i) || (ia[j][1] == i)) && (ia[j][3] > 0))
                updown++;
         }
         if ((numconns == 4)&& dupatoms(atom))
               continue;

         if ((numconns == 4) && flag3d)
         {
            cc[i-1] = 1;
            nstereo++;
         }
         if ((numconns == 4) && (updown == 2) && !flag3d)
         {
            cc[i-1] = 1;
            nstereo++;
         }
     }
     return nstereo;
}

// Remove stereo information from non-chiral carbons
int cleanconnections(int q, int nfrags, Int4 *tcon, Chr12 *tfr)
{
    int i, j, k, x, anythesame(char [4][12]);
    char conn[4][12];
```

```
    x = 0;
    for (i = 1; i <= nfrags; i++)
    {
        j = 0;
        for (k = 0; k < q; k++)
            if (tcon[k][0] == i)
               j++;
        if (j != 4)
           continue;
        j = 0;
        for (k = 0; k < q; k++)
            if (tcon[k][0] == i)
               strcpy(conn[j++],tfr[k]);
        if (!anythesame(conn))
           continue;
        for (k = 0; k < q; k++)
            if (tcon[k][0] == i)
            {
               tcon[k][3] = 0;
               if (tcon[k][1] < 9000)
                  strcpy(tfr[k],"");
               else
               {
                  strcpy(tfr[k],"X");
                  x++;
               }
            }
    }

    if (x)
    {
      for (k = 0; k < q; k++)
      {
          if (strcmp(tfr[k],"X") != 0)
             continue;
          if (k != (q-1))
          {
             for (i = k; i < q; i++)
             {
                 tcon[i][0] = tcon[i+1][0];
                 tcon[i][1] = tcon[i+1][1];
                 tcon[i][2] = tcon[i+1][2];
                 tcon[i][3] = tcon[i+1][3];
                 strcpy(tfr[i],tfr[i+1]);
             }
             k--;
          }
          q--;
      }
    }

    for (k = 0; k < q; k++)
        if (tcon[k][1] < 9000)
           strcpy(tfr[k],"");

    return q;
}
```

```
// Returns TRUE if any two of atom substituents are same (allow multiple CHs
!!!)
int anythesame(char a[4][12])
{
    int i,j;

    for (i = 0; i < 3; i++)
        for (j = i+1; j < 4; j++)
            if ((strcmp(a[i],a[j]) == 0) && (strcmp(a[i],"CH") != 0))
               return TRUE;

    return FALSE;
}

// Returns TRUE if multiple H, F, Cl, Br, I found on saturated C (=> not
chiral)
int dupatoms(char a[4][3])
{
    int i, j, numelement[5];
    static char *elements[] = { "H", "F", "Cl", "Br", "I" };

    for (i = 0; i < 5; i++)
        numelement[i] = 0;

    for (i = 0; i < 4; i++)
        for ( j = 0; j < 5; j++)
            if (strcmp(a[i],elements[j]) == 0)
               numelement[j]++;

    for (i = 0; i < 5; i++)
        if (numelement[i] > 1)
           return TRUE;

    return FALSE;
}

// Exit program
void exitprog(int freeflag)
{
     if (freeflag)
        free(molfline);
     exit(0);
}
```
