# Supplementary material for: Modular Chemical Descriptor Language (MCDL): Stereochemical modules
Source: J Cheminform. 2011 Jan 31;3:5. doi: 10.1186/1758-2946-3-5 (PMC3042968; doi:10.1186/1758-2946-3-5)
Supplement: Additional file 3 — preprocess. The source code of the C program "PREPROCESS" (version 1.0, 6 pages). [file 1758-2946-3-5-S3.PDF]

```c
/*****************************************************************/
/* preprocess.c - Preprocessor for LINDES MOL file input            */
/* Version 1.0 November 2004                                        */
/* Mike Burnett, Oak Ridge National Laboratory                      */
/*                                                                  */
/* Disclaimer of Liability                                          */
/*    This software was prepared as an account of work sponsored     */
/*    by an agency of the U.S. Government. Neither the U.S.          */
/*    Government nor any agency thereof, or any of their employees,  */
/*    makes any warranty, express or implied, or assumes any legal   */
/*    liability or responsibility for the accuracy, completeness,    */
/*    or usefulness of any information contained herein or results   */
/*    produced from application of this software, or represents that */
/*    its use would not infringe privately owned rights.             */
/*****************************************************************/

#include <stdio.h>
#include <stdlib.h>
#include <string.h>

typedef int Int4[4];
typedef char Char81[81];

Char81 *molfline;
int molflinecount, molfatoms, molfbonds;
Char81 molfilename;
int freeflag;
void exitprog(int);

main(int argc, char **argv)
{
    void ReadMolFile(void), CheckMOLFile(void);

    freeflag = 0;

    if (argc == 2)
        strcpy(molfilename,argv[1]);
    else // get user input
    {
        printf("Enter .mol file name: ");
        scanf("%s",molfilename);
    }

    if (strncmp(&molfilename[strlen(molfilename)-4],".mol",4) != 0)
    {
       printf("Input file name must end in '.mol'.\n");
       exitprog(freeflag);
    }

    ReadMolFile();
    CheckMOLFile();

    exitprog(freeflag);
}

/* Check MOL file for unconnected substructures */
void CheckMOLFile(void)
```

```
{
    int *atoms, i, j, found, numatoms, numbonds, firstbond, filenum;
    int nextatomnum, chgcount, chgarray[10][2], chargesinline;
    int unattachedatoms;
    Int4 *bonds;
    Char81 line, tfile;
    char *a;
    int getchgs(char *, int charges[10][2]);
    FILE *o_file;

    atoms = (int *) malloc(molfatoms*sizeof(int));
    bonds = (Int4 *) malloc(molfbonds*sizeof(Int4));

    a = strstr(molfilename,".mol");
    *a = '\0';

    for (i=0; i<molfatoms; i++)
        atoms[i] = 0;

    // read bond block
    for (i=0; i<molfbonds; i++)
    {
        strncpy(line,&molfline[4+molfatoms+i][0],3);
        line[3] = '\0';
        sscanf(line,"%d",&bonds[i][0]);
        strncpy(line,&molfline[4+molfatoms+i][3],3);
        line[3] = '\0';
        sscanf(line,"%d",&bonds[i][1]);
        bonds[i][2] = 0;
    }

    filenum = 0;

    while (1)
    {
        numatoms = 0;
        numbonds = 0;

        found = 0;
        for (i=0; i<molfbonds; i++)
            if (bonds[i][2] == 0)
            {
               atoms[bonds[i][0]-1] = 1;
               atoms[bonds[i][1]-1] = 1;
               bonds[i][2] = 1;
               found = 1;
               firstbond = i;
               break;
            }

        if (!found)
           break;

        while (found)
        {
              found = 0;
              for (i=firstbond+1; i<molfbonds; i++)
```

```
                  if (bonds[i][2] == 0)
                     if (atoms[bonds[i][0]-1] == 0 && atoms[bonds[i][1]-1] ==
0)
                        continue;
                     else
                     {
                        atoms[bonds[i][0]-1] = 1;
                        atoms[bonds[i][1]-1] = 1;
                        bonds[i][2] = 1;
                        found++;
                     }
        }

        for (i=0; i<molfatoms; i++)
            if (atoms[i] == 1)
               numatoms++;

        for (i=0; i<molfbonds; i++)
            if (bonds[i][2] > 0)
               numbonds++;

        // if all atoms in original MOL file connected in single structure,
exit
        if ((numatoms == molfatoms) && (numbonds == molfbonds))
        {
           printf("All atoms in original MOL file are connected in a single
structure.\n");
           break;
        }

        if (numatoms)
        {
           sprintf(tfile, "%s_%d.mol", molfilename, ++filenum);
           printf("Creating file: %s\n", tfile);
           o_file = fopen(tfile, "w");
           fprintf(o_file, "%s", molfline[0]);
           fprintf(o_file, "%s", molfline[1]);
           fprintf(o_file, "%s", molfline[2]);
           fprintf(o_file, "%3d%3d%s",numatoms,numbonds,&molfline[3][6]);
           nextatomnum = 1;
           for (i=0; i<molfatoms; i++)
               if (atoms[i] == 1)
               {
                  fprintf(o_file, "%s",molfline[4+i]);
                  atoms[i] = nextatomnum++;
               }

           for (i=0; i<molfbonds; i++)
               if (bonds[i][2] == 1)
                  fprintf(o_file, "%3d%3d%s",atoms[bonds[i][0]-
1],atoms[bonds[i][1]-1],
                          &molfline[4+molfatoms+i][6]);

           for (i=0; i<molflinecount; i++)
               if (strncmp(&molfline[i][3],"CHG",3) == 0)
               {
                  chgcount = getchgs(molfline[i],chgarray);
```

```
                  chargesinline = 0;
                  for (j=0; j<chgcount; j++)
                      if (atoms[chgarray[j][0]-1] > 0)
                         chargesinline++;
                  if (chargesinline)
                  {
                     fprintf(o_file, "M  CHG%3d", chargesinline);
                     for (j=0; j<chgcount; j++)
                         if (atoms[chgarray[j][0]-1] > 0)
                            fprintf(o_file, "%4d%4d", atoms[chgarray[j][0]-
1], chgarray[j][1]);
                     fprintf(o_file, "\n");
                  }
               }
           fprintf(o_file, "M  END\n");
           fclose(o_file);
        }

        for (i=0; i<molfatoms; i++)
            if (atoms[i] > 0)
               atoms[i] *= -1;
        for (i=0; i<molfbonds; i++)
            if (bonds[i][2] == 1)
               bonds[i][2] = -1;
    }

    // look for and list unattached atoms
    unattachedatoms = 0;
    for (i=0; i<molfatoms; i++)
        if (atoms[i] == 0)
        {
           unattachedatoms = 1;
           break;
        }
    if (unattachedatoms)
    {
       printf("The following atoms in the original MOL file\n");
       printf("are not attached to the rest of the molecule:\n");
       for (i=0; i<molfatoms; i++)
           if (atoms[i] == 0)
              printf("%d\n", i+1);
    }

    free(atoms);
    free(bonds);
    exitprog(freeflag);

}

// Extract charge information from M  CHG line
int getchgs(char *chgline, int charges[10][2])
{
    int chgcount, i, value, counter;
    char b[5];

    strncpy(b,&chgline[6],3);
    b[3] = '\0';
```

```
    sscanf(b, "%d", &chgcount);
    counter = 0;
    for (i=0; i<chgcount*2; i++)
    {
        strncpy(b,&chgline[9+i*4],4);
        b[4] = '\0';
        sscanf(b, "%d", &value);
        if (!(i%2))
           charges[counter][0] = value;
        else
           charges[counter++][1] = value;
    }

    return chgcount;
}

// Read in the lines of the MOL file
void ReadMolFile(void)
{
     FILE *i_file;
     Char81 line;
     int linecount;

     if ((i_file = fopen(molfilename,"r")) == NULL)
     {
        printf("Cannot open specified input file.\n");
        exitprog(freeflag);
     }

     // count lines in MOL file
     molflinecount = 0;
     while (fgets(line,81,i_file))
           molflinecount++;

     // allocate memory to store MOL file lines
     molfline = (Char81 *) malloc(molflinecount*sizeof(Char81));

     freeflag = 1;

     rewind(i_file);

     // read and store MOL file lines
     for (linecount=0; linecount<molflinecount; linecount++)
         fgets(molfline[linecount],81,i_file);

     fclose(i_file);

     // extract numbers of atoms and bonds in MOL file
     strncpy(line,&molfline[3][0],3);
     line[3] = '\0';
     sscanf(line,"%d",&molfatoms);
     strncpy(line,&molfline[3][3],3);
     line[3] = '\0';
     sscanf(line,"%d",&molfbonds);
}

// Exit the program
```

```
void exitprog(int freeflag)
{
     if (freeflag)
        free(molfline);
     exit(0);
}
```
